# A network of transcription factors governs the dynamics of NODAL/Activin transcriptional responses

**DOI:** 10.1242/jcs.259972

**Published:** 2022-04-26

**Authors:** Davide M. Coda, Harshil Patel, Ilaria Gori, Tessa E. Gaarenstroom, Ok-Ryul Song, Michael Howell, Caroline S. Hill

**Affiliations:** 1 Developmental Signalling Laboratory, The Francis Crick Institute, London, NW1 1AT, UK; 2 Bioinformatics and Biostatistics Facility, The Francis Crick Institute, London, NW1 1AT, UK; 3 High Throughput Screening Facility, The Francis Crick Institute, London, NW1 1AT, UK

**Keywords:** ATAC-seq, FOXI3, NODAL, Activin, SMAD, Transcription, ZIC3

## Abstract

SMAD2, an effector of the NODAL/Activin signalling pathway, regulates developmental processes by sensing distinct chromatin states and interacting with different transcriptional partners. However, the network of factors that controls SMAD2 chromatin binding and shapes its transcriptional programme over time is poorly characterised. Here, we combine ATAC-seq with computational footprinting to identify temporal changes in chromatin accessibility and transcription factor activity upon NODAL/Activin signalling. We show that SMAD2 binding induces chromatin opening genome wide. We discover footprints for FOXI3, FOXO3 and ZIC3 at the SMAD2-bound enhancers of the early response genes, *Pmepa1* and *Wnt3*, respectively, and demonstrate their functionality. Finally, we determine a mechanism by which NODAL/Activin signalling induces delayed gene expression, by uncovering a self-enabling transcriptional cascade whereby activated SMADs, together with ZIC3, induce the expression of *Wnt3*. The resultant activated WNT pathway then acts together with the NODAL/Activin pathway to regulate expression of delayed target genes in prolonged NODAL/Activin signalling conditions.

This article has an associated First Person interview with the first author of the paper.

## Abstract (precis)

**Summary:** ATAC-seq and footprinting analysis reveals the transcription factor networks underlying NODAL/Activin signalling responses and a self-enabling regulatory mechanism that explains the expression of delayed NODAL/Activin target genes.

## INTRODUCTION

During embryonic development, extracellular signals induce programmes of gene expression via activation of transcriptional effectors functioning as signal-driven transcription factors (SDTFs) (Perrimon et al., 2012). In this context, cells respond differentially to different durations of ligand exposure by constantly integrating inputs from extracellular signals with the transcriptional circuitries that define cell identity (Hagos and Dougan, 2007; Hnisz et al., 2015; van Boxtel et al., 2015). Underlying these processes are temporally coordinated changes in chromatin accessibility, where lineage-derived transcription factors (LDTFs), likely acting in concert with SDTFs, guide the chromatin remodelling machinery to specific DNA sequences (Klemm et al., 2019). Despite these insights, how the temporal dynamics of extracellular signals are interpreted at the level of chromatin to elicit changes in gene expression remains a major unanswered question.

NODAL/Activin signalling provides an excellent model system to study how cells interpret extracellular signals with respect to complex programmes of gene expression. NODAL and the highly-related ligand Activin A, which is frequently used *in vitro* to mimic the functional activities of NODAL, are members of the transforming growth factor β (TGF-β) ligand family. During embryonic development NODAL is required both for maintaining the pluripotency state in early embryos and for inducing mesendoderm differentiation and left–right patterning at later developmental stages (Arnold and Robertson, 2009; Schier, 2009; Shen, 2007). To date, the transcriptional mechanisms that enable NODAL signalling to have specific functions at different stages of differentiation are still poorly understood.

NODAL/Activin bind to heterotetrameric type I–type II serine/threonine kinase receptor complexes, which phosphorylate the receptor-regulated SMADs, SMAD2 and SMAD3, which then complex with SMAD4 to regulate gene expression in the nucleus (Pauklin and Vallier, 2015; Wu and Hill, 2009). Activated SMAD complexes have low affinity and low specificity for DNA and frequently require other transcription factors (TFs) to bind to chromatin (Gaarenstroom and Hill, 2014). Indeed, activated SMADs have been shown to interact with distinct LDTFs in different cell types, among which are FOXH1, PU.1, MYOD1, MIXER (also known as MIXL1), POU5F1 (also known as OCT4), SOX2, NANOG, EOMES and TEAD (Beyer et al., 2013; Brown et al., 2011; Chen et al., 1996; Faial et al., 2015; Germain et al., 2000; Kunwar, 2003; Mullen et al., 2011). Since many of these TFs are responsible for maintaining specific cell identities and some may act as pioneer factors, which recognize their binding sites in condensed chromatin (Brown et al., 2011; Mullen et al., 2011), the assumption has been that SMADs are recruited to already accessible chromatin by pre-bound TFs. However, we and others have shown that SMAD complexes can bind nucleosome-associated regions without the requirement for TF pioneering activity (Adam et al., 2018; Coda et al., 2017). There is also extensive interplay between the SMADs and other SDTFs, as is exemplified by the connection between TGF-β and WNT signalling during embryonic development and tissue regeneration (Oshimori and Fuchs, 2012; Reid et al., 2012; Trompouki et al., 2011). How SMAD complexes interact with different DNA binding partners to remodel chromatin, however, remains an open question.

The murine P19 embryonic teratoma cell line is ideal for analysing transcription and chromatin changes after different durations of NODAL/Activin signalling. Like embryonic stem cells (ESCs), P19s express pluripotency and mesendodermal genes in response to NODAL/Activin signalling; however, they do not differentiate when treated with the NODAL/Activin type I receptor inhibitor SB-431542 (Vallier et al., 2005). Using P19s, we have previously shown that complexes of phosphorylated SMAD2 (pSMAD2) and SMAD4 directly induce chromatin remodelling and orchestrate a complex programme of gene expression downstream of NODAL/Activin signalling. However, changes in chromatin accessibility have only been described for a small subset of target genomic loci in that study (Coda et al., 2017). Furthermore, although SMAD-recruiting TFs like FOXH1 explain chromatin opening at some genomic loci, SMADs also bind to other sites and remodel the chromatin in the absence of FOXH1. A good example of a gene thus regulated is *Pmepa1*, which is a common target of TGF-β family signalling in many different cell types (Coda et al., 2017; Levy and Hill, 2005; Watanabe et al., 2010). Finally, the key molecular players responsible for shaping the transcriptional responses after prolonged signalling have not yet been identified.

Here, we have used the assay for transposase-accessible chromatin using sequencing (ATAC-seq) followed by TF footprint analysis to discover the transcription factor networks regulating the temporal transcriptional responses to NODAL/Activin signalling. We have performed ATAC-seq on P19 cells over a time course of NODAL/Activin treatment and have integrated it with the RNA-sequencing (RNA-seq) data and SMAD2 chromatin immunoprecipitation sequencing (ChIP-seq) data that we previously generated (Coda et al., 2017). We identify distinct temporal patterns of chromatin accessibility at SMAD2-binding sites and characterise the dynamics of TF activity at these transcriptional enhancers. We then perform footprint analysis at specific loci, and discover that FOXO3, FOXI3 and ZIC3 DNA-binding motifs are necessary for NODAL/Activin-induced transcription via these particular enhancers. Finally, we address how NODAL/Activin signalling induces delayed transcriptional responses. We show that NODAL/Activin directly induces the expression of *Wnt3* via ZIC3 and FOXH1, and that WNT3 then signals via the β-catenin-TCF/LEF axis and acts with pSMAD2 to synergistically activate expression of delayed NODAL/Activin target genes. Our work thus describes an innovative approach to identify novel cofactors mediating SMAD chromatin binding and unmasks a self-enabling regulatory mechanism shaping long-term NODAL/Activin transcriptional responses.

## RESULTS

### NODAL/Activin signalling triggers genome-wide changes in chromatin accessibility at SMAD2-binding sites

Our previous work demonstrated that the complex programme of gene regulation downstream of NODAL/Activin signalling relies on chromatin remodelling events and a network of TFs that are still poorly characterised (Coda et al., 2017). To investigate the relationship between these two findings we carried out ATAC-seq in four different signalling states: inhibited (SB-431542 treated); acute (1 h Activin-treated); prolonged (8 h Activin-treated) and untreated/autocrine [chronic signalling, due to the autocrine production of NODAL and GDF3 in these cells (Coda et al., 2017)]. For each signalling condition, the read counts across two replicate experiments correlated very well with each other, and the size distribution plots of the sequenced fragments all exhibited the pattern expected (Buenrostro et al., 2013) (Fig. S1A–C).

**Fig. 1.**
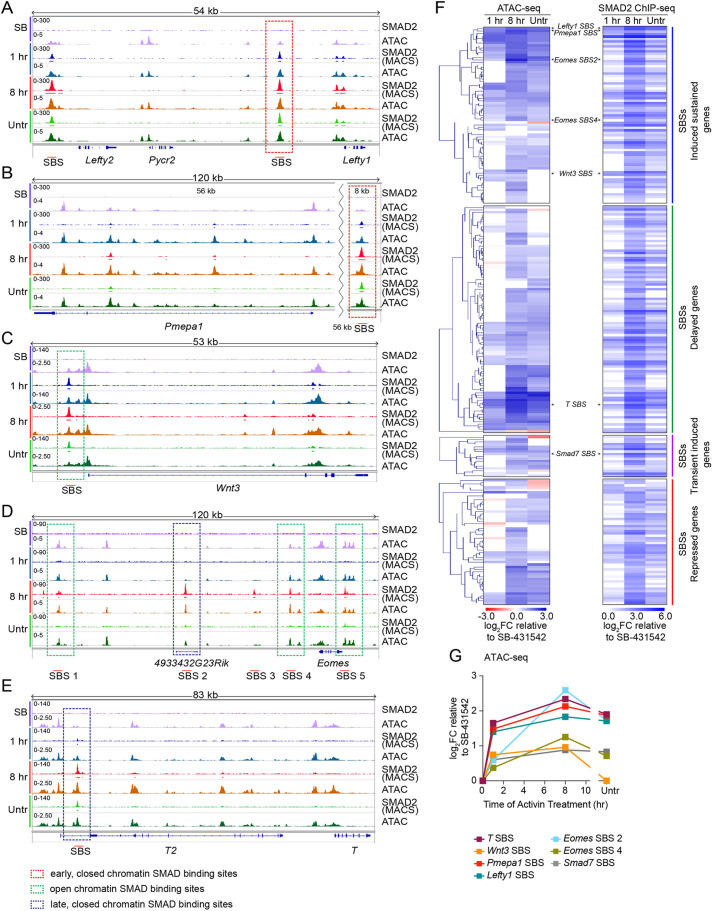
**ATAC-seq reveals genome-wide changes in chromatin accessibility at SMAD2-binding sites following NODAL/Activin signalling.** (A–E) IGV browser visualization of SMAD2 ChIP-seq and ATAC-seq experiments performed in P19 cells treated as indicated. For the SMAD2 ChIP-seq, the MACS-called peaks are also shown. The genomic loci displayed refer to the genes *Lefty1* (A), *Pmepa1* (B), *Wnt3* (C), *Eomes* (D), *T* (E). Red boxes, early SMAD2 binding to closed chromatin; green boxes, early SMAD2 binding to open chromatin; blue boxes, delayed SMAD2 binding to closed chromatin. SBS, SMAD2-binding site. (F) The SBSs positive for a DiffReps interval were divided according to the four kinetics categories of their associated genes. For each group of peaks, the heatmap on the left displays the log_2_FC relative to SB-431542 for ATAC-seq at the different time points. The order of peaks in each group was obtained by hierarchically clustering the data (left-most part of the figure). For all SBSs, the presence (blue) or absence (white) of a MACS-called SMAD2 peak at each time point is shown in the heatmap on the right. (G) The ATAC-seq data for some representative SBSs, which are indicated in F, are plotted. SB, SB-431542; 1 h, 1 h Activin; 8 hr, 8 h Activin; Untr, untreated. Results shown represent two repeats.

We first inspected the ATAC-seq data over time at some representative loci to understand how SMAD2-binding dynamics related to chromatin accessibility. *Lefty1* and *Lefty2* are two genes that were rapidly induced by Activin at the 1 h time point and whose expression was then sustained. The ATAC-seq signal was enriched at SMAD2-binding sites (SBSs) and additionally at the promoters/transcription start sites (TSSs) of *Lefty1*. Importantly, the intensity of the peaks at *Lefty1* and *Lefty2* SBSs substantially increased upon NODAL/Activin treatment compared to in the SB-431542 condition (Fig. 1A). This confirmed that these loci were in a closed conformation in the absence of signalling and opened up following SMAD2 activation. A similar scenario was also observed at the major SBS regulating *Pmepa1*, another gene expressed with the same dynamics as *Lefty1* and *Lefty2* (Fig. 1B). Of note, other ATAC-seq peaks surrounding these two genes, and not associated with SMAD2 peaks, for example those just upstream of *Pycr2*, were unchanged over the time course (Fig. 1A,B). Furthermore, in contrast to the SMAD2 peaks in the *Lefty1*, *Lefty2* and *Pmepa1* loci, the chromatin at the SBSs controlling *Wnt3*, another early induced gene, was already open in the absence of signalling and the ATAC-seq peak intensity remained fairly constant over time, as was the case for *Trh*, a delayed gene (Fig. 1C; Fig. S1D).

*Smad7* is an example of a transiently induced gene target of SMAD2 signalling*.* Here, SMAD2 binding occurred at an already accessible site, which further opened at later time points, correlating with increased SMAD2 occupancy (Fig. S1E). The ATAC-seq signal also reflected the SMAD2 occupancy in the case of repressed genes, as exemplified by the *Tbx3* locus (Fig. S1F). Importantly, we observed that distinct modes of SMAD2 binding could be identified not just in response to acute stimulation, but also at later time points. In the case of the delayed gene *Eomes*, SMAD2 binding occurred at both open (SBSs 1, 4 and 5) and closed (SBS 2) chromatin regions 8 h after Activin treatment (Fig. 1D). A similar scenario was observed in the case of the gene *T* (also known as *Tbxt*) (Fig. 1E). We thus identified three modes of SMAD2 binding – early binding to closed chromatin (SBSs marked in red); early binding to open chromatin (SBSs marked in green); and delayed binding to closed chromatin (SBSs marked in blue) (Fig. 1A–E).

To substantiate the biological relevance of our study, we compared the SMAD2 ChIP-seq data obtained in P19 cells with those generated by the Massagué laboratory in mouse embryoid bodies treated with Activin (Aragon et al., 2019). Indeed, the SMAD2 ChIP-seq peaks were completely conserved at all the genomic loci we focus on in this study (Fig. S2). Of note, the enhancers for *Nodal* and *Lefty2* occupied by SMAD2 in response to Activin in P19 cells are also the same ones previously described as NODAL-responsive enhancers in mouse embryos (data not shown; Saijoh et al., 2000).

Next, we identified regions of enriched ATAC-seq signal at the genome-wide level for each of the four conditions using the MACS.2 software. The total number of intervals identified and their annotation according to the different genomic features were almost identical across samples, and for both acute and prolonged NODAL/Activin signalling >90% of the consensus SBSs overlapped an ATAC-seq peak by at least 1 bp. Surprisingly this proportion was also maintained in the SB-431542 state (Fig. S3A,B), suggesting that even though many SBSs, such as those regulating *Lefty1* and *Pmepa1*, are occupied by nucleosomes in absence of signalling (Coda et al., 2017), additional features must mark these sites making them slightly more accessible to the transposase Tn5 compared to the surrounding chromatin.

For further analysis, we created a dataset of consensus ATAC-seq peaks by merging the peaks from the different time points. As expected, the consensus ATAC-seq intervals intersected with 456 out of 478 high confidence SMAD2 consensus peaks previously defined (Coda et al., 2017). Importantly, the large majority of these 456 intervals was entirely encompassed by a consensus ATAC-seq peak, and all of them shared greater than 40% their length in common (Fig. S3C). We then quantified changes in chromatin accessibility over time at the high confidence set of SMAD2 binding sites, using the DiffReps package to achieve statistical relevance (Shen et al., 2013). For the high confidence set of SMAD2 peaks, we identified DiffReps intervals of differential ATAC-seq at 236 sites out of 478, meaning that in at least one signalling condition the chromatin accessibility at these loci changed compared to the SB-431542 state (Fig. S3D). The SBSs that showed temporal changes in chromatin accessibility were grouped according to the kinetic category of the genes they were associated with, and the temporal changes in ATAC-seq were displayed in a heat map adjacent to the time at which the SMAD2 peak was detected at each site (Fig. 1F; Table S1). Overall, the chromatin accessibility of SBSs tended to increase upon NODAL/Activin signalling, regardless of the dynamics of induction/repression of the associated target gene (Fig. 1F). Moreover, the ATAC-seq changes correlated well with the time of SMAD2 binding, confirming that SMAD complexes directly elicited the chromatin remodelling of their target sites. In fact, for the majority of SBSs, no differences in the ATAC-seq levels were detected after 1 h Activin stimulation compared to the SB-431542 condition, but the chromatin accessibility of these loci increased with prolonged Activin signalling, coincident with SMAD2 binding (Fig. 1F,G). Nevertheless, when a SMAD2 peak was detected upon acute stimulation, the ATAC-seq signal over the corresponding SBSs also increased. The quantitative data displayed in the heatmap also confirmed what we first observed by visual inspection. For example, the ATAC-seq signal at the *Lefty1* and *Pmepa1* SBSs significantly increased upon pathway activation compared to in the SB-431542 state (Fig. 1G). Similarly, the ATAC-seq changes over time as quantified by DiffReps for the *Eomes* and *Smad7* SBSs were also in agreement with the IGV screenshots (Fig. 1G).

In our previous analysis of nucleosome occupancy during the same NODAL/Activin time course we showed that SMAD pathway activation induced nucleosome displacement at a subset of sites associated with so-called ‘baseline off’ genes (defined as genes that were not expressed in the SB-431542 condition) (Coda et al., 2017). When comparing the SBSs associated with temporal changes in chromatin accessibility with those SBSs that were not, we noticed that the number of ‘baseline off’ SBSs in the former was statistically significantly higher than in the latter. Thus, signalling-induced changes in chromatin accessibility were more likely to occur at SBSs associated with ‘baseline off’ genes rather than ‘baseline on’ SBSs (Fig. S3E).

In conclusion, this quantitative analysis shows that SMAD2 binding directly increased chromatin accessibility at many target sites. These chromatin remodelling events do not exclusively occur at loci in a closed conformation, but also occur at many SBSs that are already partly open in the absence of signalling.

### Footprint analysis reveals that members of the FOX family act as SMAD cofactors for binding to closed chromatin at the *Pmepa1* SBS

**Fig. 2.**
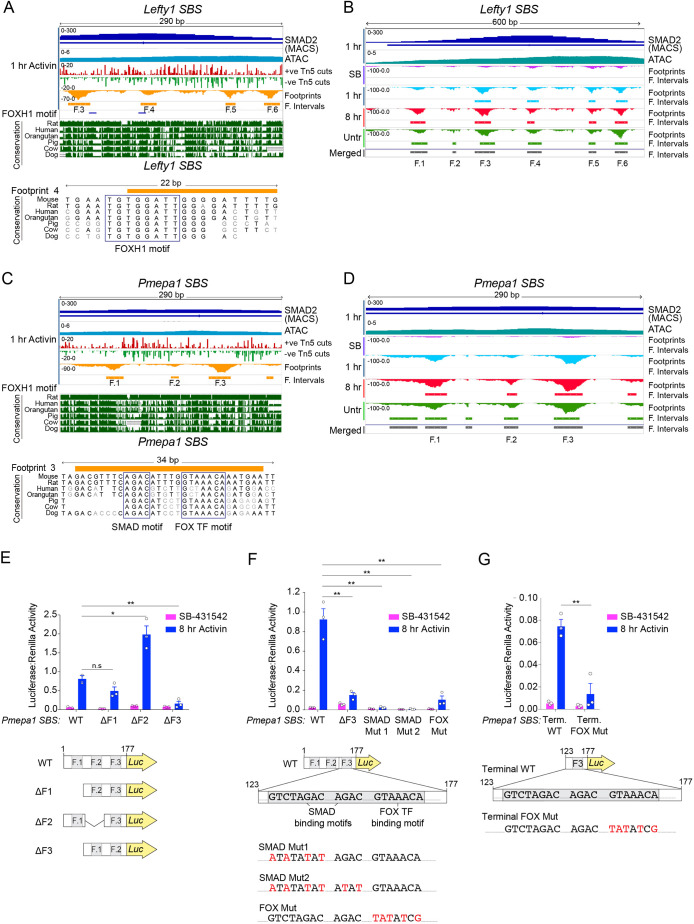
**Locus-specific footprint analysis identifies novel cofactors for SMAD binding to closed chromatin.** (A) Screenshot from the IGV genome browser of the *Lefty1* SBS locus for the 1 h Activin sample. Displayed are the SMAD2 ChIP-seq and relative peak interval (MACS) (dark blue), together with the track from the ATAC-seq experiment (light blue). In red and green, per nucleotide Tn5 cutting activity. In orange, the Wellington footprint prediction, and relative intervals (footprints F.3–F.6). DNA sequences matching the FOXH1 motif are denoted in dark blue. The conservation tracks downloaded from UCSC genome browser for the species indicated are also displayed. Bottom panel, zoom of the region surrounding Footprint F.4. The blue box indicates the mouse sequence matching the FOXH1 binding motif and its alignment with the species shown in the panel above. (B) Screenshot from the IGV genome browser of the *Lefty1* SBS locus. Shown are the SMAD2 ChIP-seq and relative peak interval (MACS) together with the track for the ATAC-seq experiment for the 1 h Activin sample. Underneath, the Wellington footprint predictions are displayed for each condition/time point. In grey, the consensus intervals (F.1–F.6) generated by merging the individual footprint from each sample. (C) As for A, but for the *Pmepa1* SBS locus for the 1 h Activin sample. Note there are no FOXH1 binding sites in the *Pmepa1* SBS. (D) As for B, but for the *Pmepa1* SBS locus. Results shown in A–D represent two repeats. (E,F) Luciferase activity upon Activin induction in P19 cells transfected with either the full-length *Pmepa1* SBS (1–177) reporter, or with versions of the reporter where individual footprints have been deleted (E) or where particular motifs in footprint F.3 have been mutated (F). TK-Renilla was transfected as an internal control. (G) Luciferase activity upon Activin induction in P19 cells transfected with a luciferase reporter containing only footprint F.3 or a derivative where FOX-binding site has been mutated. TK-Renilla was transfected as an internal control. In E–G, means±s.e.m. of three independent experiments performed in duplicate are plotted, with the ratio of Luciferase:Renilla shown. *P<0.05; **P<0.01; ns, not significant (unpaired two-tailed *t*-test).

Given that DNA sequences directly bound by proteins are protected from transposase activity, ATAC-seq has been successfully used to infer loci occupied by DNA-binding proteins genome-wide (Buenrostro et al., 2013; Yan et al., 2020). To unveil the network of TFs responsible for recruiting SMAD complexes to chromatin and for shaping the transcriptional responses downstream we used the Wellington tool, which does not require an *a priori* set of motifs and thus provided us with the additional advantage of identifying *de novo* TF–DNA interactions (Piper et al., 2015). The IGV screenshot for the *Lefty1* SBS illustrates the output of the footprint analysis (Fig. 2A). We used this SBS as a control for the method, as we have previously demonstrated that FOXH1 binds this site and is crucial for NODAL/Activin-induced *Lefty1* expression (Coda et al., 2017). At the *Lefty1* SBS, four footprints (named F.3–F.6) were detected 1 h after Activin treatment, and notably, two of them colocalised with FOXH1 motifs. In particular, the second footprint (F.4) was both highly conserved and positioned directly under the SMAD2 peak (Fig. 2A). Importantly, no intervals of decreased cutting density were found across the *Lefty1* SBS locus in the non-signalling condition (SB-431542), indicating that this SBS was devoid of any bound TFs in the absence of signalling (Fig. 2B). In contrast, the 1-h Activin footprints described in Fig. 2A were detected in all the other conditions (Fig. 2B). We merged the footprints identified in each individual sample to create a consensus list of intervals to use in downstream analyses for identifying temporal changes in footprint occurrences. Considering the intervals identified by Wellington alongside the phylogenetic conservation tracks provides a good strategy for identifying novel TFs that recruit SMADs to chromatin.

We next applied this approach to the *Pmepa1* SBS, as we had previously shown that SMAD2 binds to this closed site in a FOXH1-independent manner, which suggested the binding of a yet unidentified SMAD2 cofactor at this genomic locus (Coda et al., 2017). Upon acute Activin treatment three footprints were detected at conserved sites in close proximity to the SMAD2 peak summit that were absent in the SB-431542 condition (Fig. 2C,D). To validate the relevance of the individual footprints, the sequences encompassing the footprints and its mutated versions were cloned into luciferase reporters and tested for their activity in P19 cells following Activin stimulation. Luciferase induction was completely lost upon deletion of the sequences corresponding to footprint F.3, but was not affected by removing F.1 or F.2 (Fig. 2E). Closer inspection of F.3 revealed the presence of three canonical SMAD-binding elements (SBEs, AGAC/GTCT) in close proximity to a 7-bp sequence – GTAAACA – which is recognized by members of the forkhead (FOX) family of transcription factors (Nakagawa et al., 2013) (Fig. 2F). When the SMAD-binding motifs were mutated, luciferase induction following Activin treatment was lost. However, loss of luciferase activity was also greatly reduced when the GTAAACA motif was mutated, indicating that factors recognizing this element were also required for transcriptional activation (Fig. 2F). Furthermore, the sequence encompassing F.3 was sufficient for Activin-induced transcriptional activation and this was entirely dependent on an intact GTAAACA sequence (Fig. 2G).

**Fig. 3.**
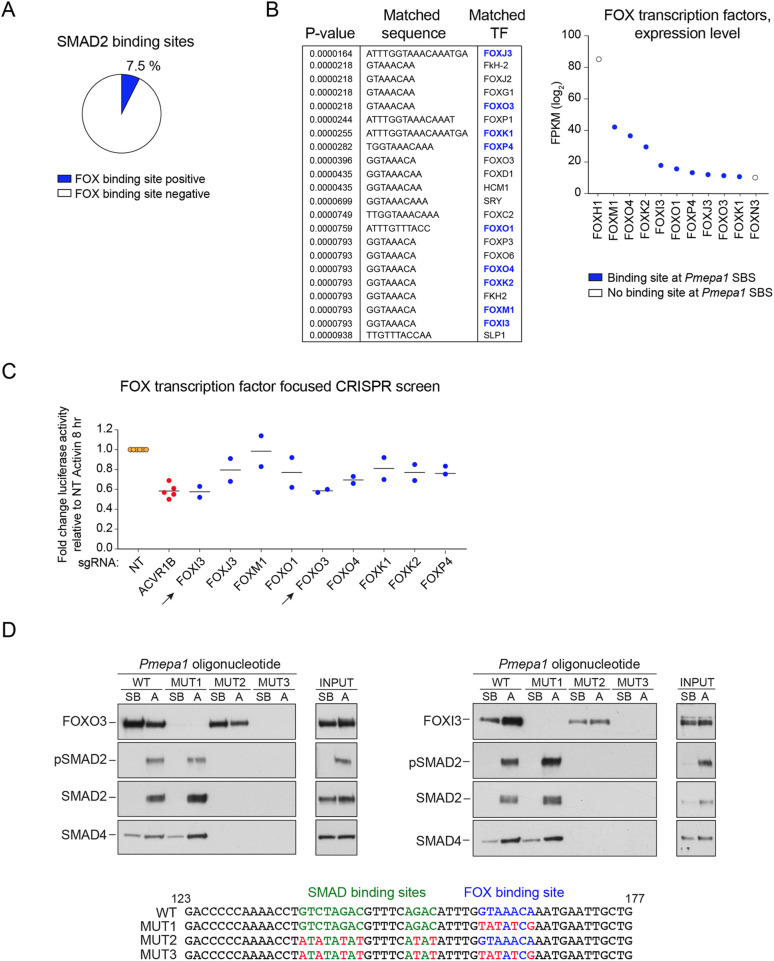
**FOXO3 and FOXI3 are required for Activin-induced induction via the *Pmepa1* SBS.** (A) The proportion of SMAD2 SBSs that contain a FOX binding site (GTAAACA motif). (B) FIMO analysis on the DNA sequence from footprint F.3 from the *Pmepa1* SBS showing the TFs whose DNA-binding motifs significantly match to the input sequence (left panel). Blue highlights the TFs belonging to the Fox family. Right panel, mRNA expression level of at least 10 FPKM (log2) in P19 cells. (C) Luciferase activity upon Activin induction in P19 cells expressing the *Pmepa1* SBS WT reporter and electroporated with sgRNA-Cas9 complexes against the Activin type I receptor *Acvr1b*, the TFs identified in A or a non-targeting (NT) sgRNA as a control. TK-Renilla was transfected as an internal control. Plotted are fold change in Luciferase:Renilla relative to the NT values at 8 h post Activin induction. Arrows indicate genes which inhibit Activin-induced transcription when knocked out individually. (D) DNAP analysis to demonstrate the binding of activated SMADs with FOXO3 (left panels) or FOXI3 (right panels) to the footprint F.3 of the *Pmepa1* SBS and mutants thereof. Nuclear extracts were prepared from WT P19 cells that had been incubated with 10 μM SB-431542 (SB) overnight, washed out and treated with either SB-431542 or 20 ng/ml Activin (A) for 1 h. The extracts were analysed in a DNAP assay using the biotinylated oligonucleotides shown. The western blots were probed with the antibodies indicated and the inputs are shown on the right of the DNAPs. Results shown represent three repeats.

The presence of a FOX binding site was not unique to the *Pmepa1* enhancer, but occurred in 7.5% of SMAD2 SBSs (Fig. 3A). To determine the identity of the FOX family member(s) responsible for Activin-induced transcriptional activation via the *Pmepa1* SBS we performed a focused CRISPR/Cas9 screen with single guide (sg)RNAs against members of the FOX family most highly expressed in P19 cells (Fig. 3B) using the luciferase reporter driven by the sequences corresponding to footprints F.1–F.3 as a readout. The screen identified FOXO3 and FOXI3 as TFs required for Activin-induced transcription of this reporter (Fig. 3C). Indeed, CRISPR/Cas9-mediated knockout of FOXI3 and FOXO3, but not of any of the other candidates, resulted in a decrease in luciferase activity following Activin treatment that was comparable to that achieved by targeting an essential component of the signalling cascade, the Activin type I receptor ACVR1B (Fig. 3C).

We went on to validate this finding biochemically using a DNA pulldown (DNAP) assay. We found that both FOXO3 and FOXI3 bound the footprint sequence in the presence and absence of Activin stimulation, and this did not require the presence of intact neighbouring SBEs (Fig. 3D). Similarly, although SMAD binding required intact SBEs, it did not depend on the binding of FOXO3 or FOXI3 (Fig. 3D). Thus, the FOX proteins and activated SMADs bind to their cognate sites independently of each other, but both are required for transcriptional activity.

Taken together, these experiments identify FOXI3 and FOXO3 as SMAD2 cofactors required for binding to closed chromatin enhancers, such as the *Pmepa1* SBS, inducing chromatin remodelling and activating transcription.

### Chromatin accessibility at TCF/LEF sites increases in prolonged NODAL/Activin signalling conditions

Having validated the predictive power of the Wellington algorithm at selected SMAD2 target loci, we aimed to use it genome-wide to visualise how footprints change over time in response to NODAL/Activin signalling. We used the transcriptional regulator CTCF as a proof of principle, as it generates footprints that are readily detectable with ATAC-seq data (Buenrostro et al., 2013). As expected, we observed a stereotypical CTCF footprint comparable to those previously obtained from ATAC-seq experiments in other cell lines (Buenrostro et al., 2013), with no apparent differences across the different samples (Fig. S4A). When we performed the same analysis with FOXH1 genome wide, we could readily detect a footprint at a subset of loci (Fig. S4B). However, when we repeated the analysis to understand how occupancy of FOXH1 sites that reside in SMAD2 peaks changed over time, the heatmaps showed a much less well-defined pattern (Fig. S4C). Thus, despite the fact that the FOXH1-binding site in the *Lefty1* SBS is part of a larger footprint (Fig. 2A,B), when all the FOXH1 sites in the SMAD2 SBSs are compared, a discrete footprint is not evident because Tn5 can cut in the centre of the motif. This suggests that like many TFs, FOXH1 has a short residence time on the DNA, and thus leaves a poorly detectable footprint due to a rapid cycling on and off chromatin (Baek et al., 2017; Swinstead et al., 2016).

**Fig. 4.**
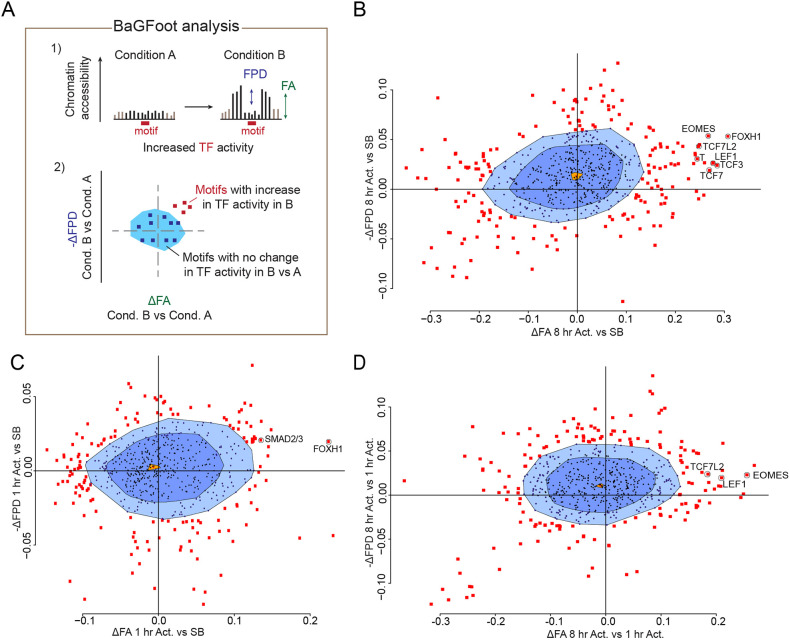
**BaGFoot analysis identifies the transcription factor network downstream of NODAL/Activin signalling.** (A) A scheme showing the BaGFoot analysis. TF activation elicits alteration of chromatin accessibility that can be measured as changes in openness within the motif and in the sequences flanking it. Following a biological signal, changes in FPD and FA are measured for every motif genome-wide, and Δ values are given. Values for all motifs are plotted in a bag plot. The population median is marked in light orange. The bag area (dark blue) is the region where 50% of the population is located. The fence area (light blue) is the region where most (typically 97%–99%) motifs are located. (B–D) BaGFoot plots showing ΔFA and ΔFPD for the ATAC-seq peaks overlapping the SMAD2 peaks in P19 cells following different treatment conditions as follows: 8 h Activin compared to SB-431542 (B), 1 h Activin compared to SB-431542 (C), 8 h Activin compared to 1 h Activin (D). Results shown represent two repeats.

We therefore turned to a different algorithm for investigating footprints over time and were particularly interested in TFs that were responsible for activating the delayed NODAL/Activin-induced genes. Upon capturing two TF-dependent effects on chromatin simultaneously, measured as changes in TF ‘footprint depth’ (FPD) and footprint accessibility (FA) of flanking motifs (Fig. 4A), ‘bivariate genomic footprinting’ (BaGFoot) analysis has been successfully used to reveal the activity of TFs even when an absolute footprint cannot be detected (Baek et al., 2017; Braun et al., 2021). We therefore applied the BaGFoot algorithm to the ATAC-seq data in each signalling condition compared to the control (SB-431542), to assess signalling-induced changes in global TF activity. Importantly, SMAD2- and SMAD3-binding sites were among the significant outliers from the multivariate distribution showing increased TF binding after a 1-h Activin stimulation compared to in the absence of signalling (Fig. S5). To identify candidate TFs responsible for recruiting SMAD2 to specific enhancers in response to NODAL/Activin signalling, we repeated the analysis on the 456 SMAD2 peaks overlapping with an ATAC-seq peak. The known SMAD-interacting cofactors FOXH1 showed increased activity at both time points compared to the SB-431542 condition (Fig. 4B,C). Strikingly, the TCF/LEF family of TFs (LEF1, TCF3, TCF7 and TCF7L2), were only activated after 8 h of Activin treatment as measured by increases in FPD and FA, suggesting that they could be required for recruiting SMADs at delayed target genes, where we only see SMAD2 ChIP-seq peaks at the 8-h time point (Fig. 4B–D). We therefore set out to address the mechanism behind the increase in TCF/LEF occupancy in prolonged signalling conditions and its relevance for the NODAL/Activin responses.

### NODAL/Activin-dependent *Wnt3* induction is required to activate a subset of delayed target genes

**Fig. 5.**
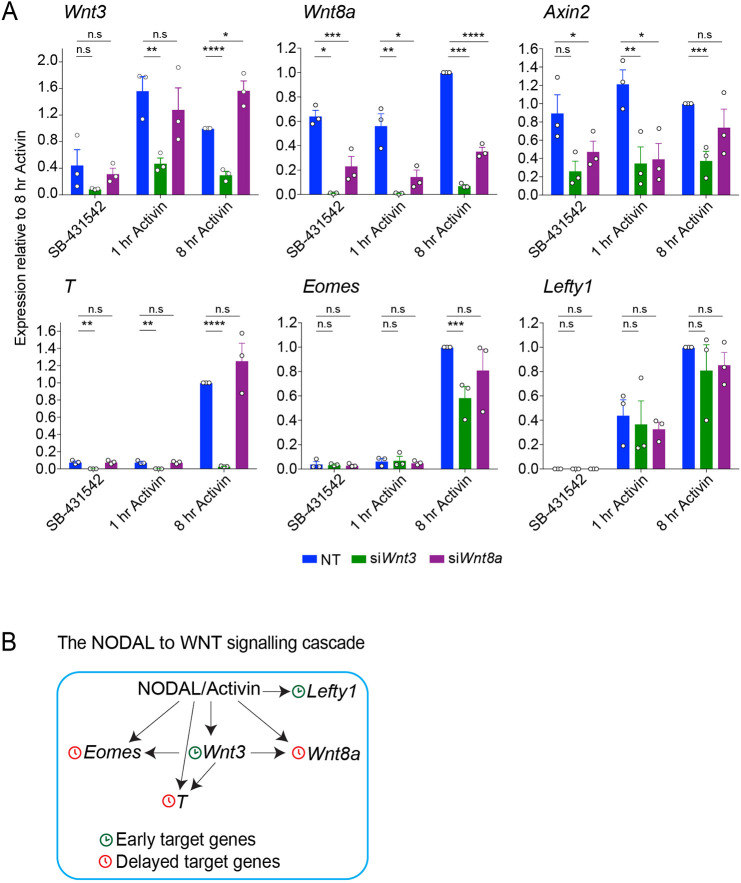
***Wnt3*, but not *Wnt8a*, is required for the full induction of NODAL/Activin delayed target genes *Eomes* and *T.*** (A) P19 cells were transfected with siRNAs directed against either *Wnt3* or *Wnt8a*, along with a non-targeting control (NT). Cells were treated overnight with SB-431542, washed out, then either treated with SB-431542 for 1 h or with Activin for the indicated times. qPCR was performed for the genes shown. Plotted are the mean±s.e.m. of three independent experiments performed in triplicate of gene expression values normalised to endogenous *Gapdh* values. *P<0.05; **P<0.01; ***P<0.001; ****P<0.0001; n.s., not significant (unpaired two-tailed *t*-test). (B) Scheme showing the relationship between NODAL and WNT signalling in P19 cells. The arrows from NODAL/Activin signalling refer to the presence of SMAD2 on the regulatory sequences of the genes in question. The arrows from *Wnt3* refer to the dependence of target gene expression on signalling from WNT3.

We first asked whether TCF/LEF expression levels were changed upon NODAL/Activin treatment by interrogating the matched RNA-seq data (Coda et al., 2017), but found that this was not the case (data not shown). Since these TFs form complexes with β-catenin and activate transcription through the WNT/β-catenin signalling cascade, we speculated that the increase in TCF/LEF chromatin binding could be ascribed to activation of the WNT/β-catenin axis, rather than being the result of an increase in TCF/LEF expression (Langton et al., 2016; Nusse and Clevers, 2017; Steinhart and Angers, 2018). Indeed, we noticed that genes encoding two members of the WNT ligand family, *Wnt3* and *Wnt8a*, were upregulated in response to NODAL/Activin signalling, although with different dynamics. *Wnt3* was directly induced immediately after 1 h of Activin, whereas *Wnt8a* mRNA was only upregulated after 8 h of signalling (Fig. 5A). This is consistent with SMAD2 ChIP-seq peaks being present at the *Wnt3* locus at 1 h, but at the *Wnt8a* locus only at 8 h (Fig. 1C; data not shown). To disentangle the individual contribution of WNT3 and WNT8 to the induction of NODAL/Activin target genes, we performed knockdown experiments. Knocking down *Wnt3*, but not *Wnt8a* impaired the induction of delayed NODAL/Activin target genes *T* and *Eomes*, but did not affect the induction of *Lefty1*, which is an immediate early NODAL/Activin target gene (Fig. 5A). Moreover, *Wnt8a* expression also depended on WNT3, as was the case for *Axin2*, which we included as it is a well-known target of WNT/β-catenin signalling (Jho et al., 2002) (Fig. 5A).

These observations suggested that early NODAL/Activin-dependent *Wnt3* expression would drive β-catenin pathway activation, which would then synergise with the NODAL/Activin pathway to induce delayed target gene expression (Fig. 5B). To test this idea, P19 cells were treated with the small-molecule inhibitors of the WNT/β-catenin signalling pathway LGK974 and IWR-1 (Nusse and Clevers, 2017), in the context of a NODAL/Activin time course. Treatment with LGK974 or IWR-1 recapitulated what was observed by knocking down *Wnt3*. The induction of *Eomes* and *T* in response to NODAL/Activin was severely compromised, particularly with the addition of LGK974, but this was not the case for *Lefty1* (Fig. S6A). *Wnt8a* and the WNT-target gene *Axin2* expression was also affected by the treatment with LGK974 and IWR-1 (Fig. S6A), consistent with our siRNA knockdown data. As expected, these inhibitors had no effect when combined with the WNT pathway activator, CHIR99021 (CHIRON; An et al., 2010), which acts downstream of them (Fig. S6A). Finally, to address to what extent the NODAL/Activin pathway synergised with the WNT/β-catenin cascade, P19 cells were treated CHIRON along with Activin, or with SB-431542 (to inhibit NODAL/Activin signalling and allow us to observe the effect of CHIRON in the absence of Activin signalling). Both *T* and *Wnt8a* were induced with similar dynamics when treated with Activin or with CHIRON in the presence of SB-431542, suggesting that both WNT and NODAL/Activin signalling cascades targeted these genes independently (Fig. S6B). Furthermore, *T* expression was greatly increased when cells were simultaneously exposed to both Activin and CHIRON, whereas this was not the case for *Lefty1*, *Eomes* or *Wnt3*, indicating the existence of gene-specific responses to different levels of activation of the WNT pathway (Fig. S6A,B). Finally, as expected, induction of the WNT target genes *Axin2* and *Notum* was almost entirely independent of NODAL/Activin signalling (Fig. S6B).

Overall, these experiments provide evidence of a temporal cascade in which the NODAL/Activin-dependent induction of *Wnt3* triggers the activation of the WNT/β-catenin pathway, which is necessary for the full induction of delayed NODAL/Activin target genes like *Eomes*, *T* and *Wnt8a* (Fig. 5B). Since we find TCF-binding sites at the enhancers of these delayed genes where we see SMAD2 recruitment only at the later 8-h timepoint, we hypothesise that the TCF/β-catenin complex synergises with the SMAD complex at these enhancers to activate this delayed transcription.

### The transcription factor ZIC3 is responsible for *Wnt3* induction in response to NODAL/Activin signalling

**Fig. 6.**
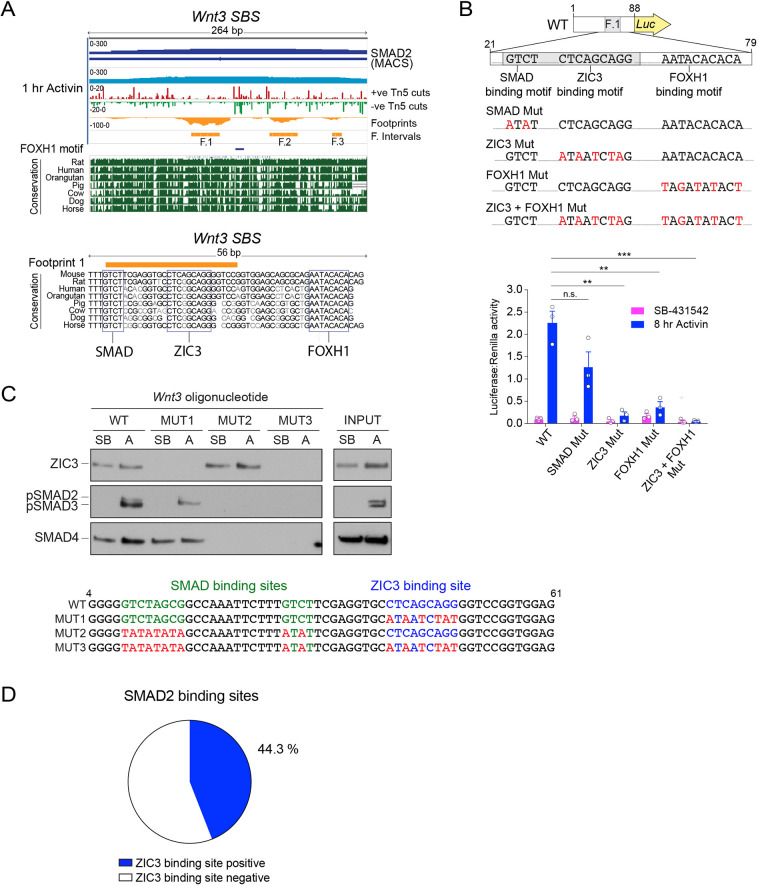
**ZIC3 and FOXH1 are required for NODAL/Activin-induced expression of *Wnt3*.** (A) Top panel, screenshot from the IGV genome browser of the *Wnt3* SBS locus for the 1 h Activin sample. Displayed are the SMAD2 ChIP-seq and relative peak interval (MACS) (dark blue), together with the track from the ATAC-seq experiment (light blue). In red and green, per nucleotide Tn5 cutting activity. In orange, the Wellington footprint prediction, and relative intervals (footprints F.1–F.3). DNA sequences matching the FOXH1 motif are denoted in dark blue. The conservation tracks downloaded from UCSC genome browser for the species indicated are also displayed. Bottom panel, zoom of the region surrounding Footprint F.1. The blue boxes indicate the mouse sequence matching the SMAD, ZIC3 and FOXH1 binding motifs and their alignment with the species shown in the panel above. (B) Luciferase activity upon Activin induction in P19 cells transfected with either the full length *Wnt3* SBS reporter, or with versions of the reporter where individual sequences were mutated as shown below. TK-Renilla was transfected as an internal control. Mean±s.e.m. of three independent experiments performed in duplicate are plotted, with the ratio of Luciferase:Renilla shown. **P<0.01; ***P<0.001; n.s., not significant (unpaired two-tailed *t*-test). (C) DNAP analysis to demonstrate the binding of ZIC3 and activated SMAD complexes to the footprint F.1 of the *Wnt3* SBS and mutants thereof. Nuclear extracts were prepared from P19 cells that had been incubated with 10 μM SB-431542 overnight, washed out and treated with either SB-431542 (SB) or 20 ng/ml Activin (A) for 1 h. The extracts were analysed in a DNAP assay using the biotinylated oligonucleotides shown. The western blots were probed with the antibodies indicated and the inputs are shown on the right. (D) The proportion of SBSs that contain a ZIC3-binding site (CNCAGCWGG). Results shown in D represent three repeats.

Finally, we set out to understand how the *Wnt3* gene itself is regulated in response to NODAL/Activin signalling. We noticed that in the first of three footprint intervals identified by Wellington analysis at the *Wnt3* promoter, there was a SMAD-binding motif adjacent to a 9-bp sequence recognized by the TF ZIC3, followed by a FOXH1 motif outside the footprint interval (Ali et al., 2012; Yang et al., 2019) (Fig. 6A). To functionally verify the relevance of these motifs, the 100-bp interval encompassing the footprint, and mutated versions thereof, were cloned into luciferase reporters and tested for their activity in P19 cells following Activin stimulation. Mutating the ZIC3 motif or the FOXH1 motif resulted in a complete loss of luciferase induction, whereas mutating the adjacent SMAD-binding site did not significantly diminish the responses measured upon Activin treatment (Fig. 6B). The latter result was probably due to additional SMAD sites upstream (Fig. 6C). Using DNAP assays we readily detected ZIC3 binding to this enhancer sequence both in the presence and absence of Activin stimulation, and this was not affected by the ability of the SMAD complexes to bind (Fig. 6C). In turn, the presence of ZIC3 did not appear to be required for SMAD binding, although, as shown above, ZIC3 is required for transcriptional activation (Fig. 6C). Taken together, these results indicate that both ZIC3 and FOXH1, and the activated SMAD complexes are required to induce *Wnt3* expression in response to Activin. Furthermore, an analysis of the occurrence of ZIC3-binding sites in SMAD2 peaks revealed that more than 40% of them contained a ZIC3 site, suggesting that its involvement in NODAL/Activin-induced transcription is not unique to *Wnt3* (Fig. 6D).

**Fig. 7.**
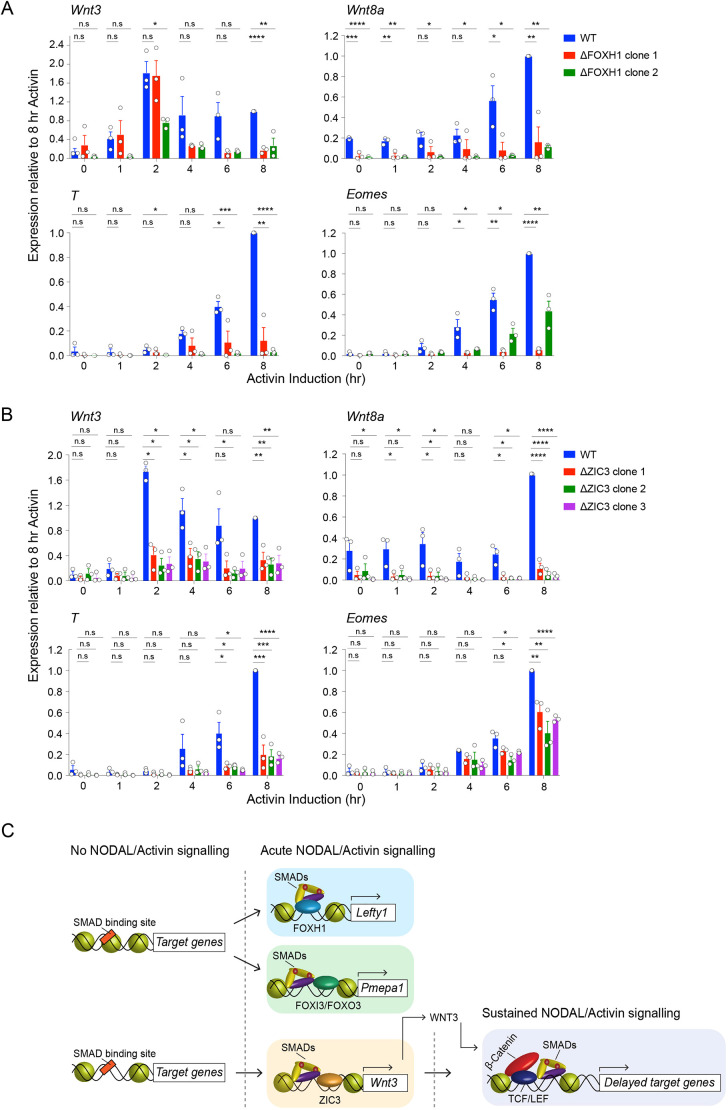
**ZIC3, but not FOXH1 is required for *Wnt3* induction.** (A,B) A time course of Activin induction was performed on two independent clones with deletions for FOXH1, and on WT P19 cells as a control (A). The same experiment was carried-out on three independent clones with deletions for ZIC3, and on WT P19 cells as a control (B). Transcript levels of representative target genes were measured by qPCR relative to *Gapdh* and plotted compared to 8 h of Activin treatment for the WT sample. Plotted are the mean±s.e.m. of three independent experiments performed in duplicate. *P<0.05; **P<0.01; ***P<0.001; ****P<0.0001; n.s., not significant (unpaired two-tailed *t*-test). (C) Schematic to show how different transcription factors recruit activated SMAD complexes to induce the activation of early and delayed NODAL/Activin targets. FOXH1 and FOXO3 or FOXI3 bind with the activated SMAD2/3–SMAD4 complexes to open up the chromatin and induce transcription of *Lefty1* and *Pmepa1*, respectively. ZIC3 binds with activated SMAD2/3–SMAD4 complexes to the *Wnt3* enhancer to induce expression of *Wnt3*. In turn, WNT3 signals to the enhancers of delayed target genes such as *Eomes* and *T* and induces their expression by cooperating with NODAL/Activin signalling at the level of their enhancers as shown.

To assess the importance of ZIC3 and FOXH1 for *Wnt3* induction in response to Activin stimulation, we knocked out ZIC3 or FOXH1 in P19 clones using CRISPR/Cas9 (Fig. S7). Clones were screened by genomic sequencing and the low levels of *Foxh1* or *Zic3* mRNA were used as a bona fide indicator of the lack of functional proteins, alongside the loss of expression for well-established downstream target genes (Fig. S7). As expected from our previous work, all clones with a FOXH1 loss-of-function mutation failed to express *Lefty1* in response to Activin, whereas *Pmepa1* induction was not affected (Fig. S7A,B) (Coda et al., 2017). Furthermore, no statistical difference in the induction of *Lefty1* or *Pmepa1* was observed in the ZIC3-null clones compared to wild type (WT) (Fig. S7C,D). Importantly, loss of FOXH1 or ZIC3 function across multiple clones resulted in a strong reduction of *Wnt3* transcription after 2 h of Activin signalling, as well as after prolonged signalling (Fig. 7A,B). We then considered the effect of *Wnt3* loss on the transcriptional cascade downstream of the WNT/β-catenin pathway. As predicted, target genes completely dependent on WNT signalling, such as *T* and *Wnt8a*, failed to be induced in response to Activin when ZIC3 or FOXH1 were deleted (Fig. 7A,B). *Eomes* induction in response to Activin in contrast was partially affected in clones of P19 cells lacking functional ZIC3 or FOXH1 (Fig. 7A,B).

We therefore conclude that ZIC3 is required for *Wnt3* induction in response to NODAL/Activin signalling, and that this is due to direct transcriptional regulation occurring at the level of the *Wnt3* enhancer, where ZIC3 binds together with FOXH1 and activated pSMAD2–SMAD4 complexes.

## DISCUSSION

### A network of transcription factors cooperates with activated SMAD complexes to induce transcription

Here, we have revealed a temporal network of TFs that underlies SMAD2 binding to chromatin and mediates its transcriptional activity over time. By combining ATAC-seq with SMAD2 ChIP-seq and RNA-seq, we provide major new insights into how a single SDTF executes a complex programme of gene expression. First, we have shown that NODAL/Activin signalling triggers global changes in chromatin accessibility that temporally correlate with SMAD2 genome binding. Focusing on enhancers bound by SMAD2 after 1 h of NODAL/Activin signalling, such as those regulating early expression of *Pmepa1* and *Wnt3*, analyses of TF footprint dynamics revealed DNA motifs bound by hitherto uncharacterised SMAD cofactors. We have demonstrated that DNA sequences recognized by FOXO3 and FOXI3 and by ZIC3 are necessary for NODAL/Activin-induced transcription via *Pmepa1* and *Wnt3* enhancers, respectively (Fig. 7C). We additionally shed light on the mechanism of delayed transcriptional responses downstream of NODAL/Activin. We show that induction of *Wnt3* by NODAL/Activin leads to activation of the WNT signalling pathway, which converges on chromatin with the activated SMADs to co-regulate genes like *Eomes* and *T*, which exhibit a delayed response to NODAL/Activin signalling (Fig. 7C). We anticipate that the network of transcription factors identified here will be a general feature of NODAL/Activin signalling, as the SBSs we study are fully conserved in mouse embryoid bodies treated with Activin to differentiate them into mesendoderm. Furthermore, *Pmepa1* is induced by TGF-β signalling in many different cell lines (Brunschwig et al., 2003; Levy and Hill, 2005; Watanabe et al., 2010), and NODAL/Activin induces *Wnt3* in both mouse and human ESCs (Aragon et al., 2019; Estaras et al., 2017; Gadue et al., 2006; Senft et al., 2018).

### Footprinting as a means of identifying bound TFs in different signalling conditions

How TFs recognize binding sites in closed chromatin is still poorly understood (Choukrallah and Matthias, 2014). We previously demonstrated for a subset of target genes that SMAD complexes do not require FOXH1 for their ability to bind to closed chromatin in response to NODAL/Activin signalling and induce chromatin remodelling (Coda et al., 2017). Here, using genome-wide ATAC-seq, we have confirmed that activated SMAD2 targets both inactive and active chromatin. At the former, SMAD2 induces *de novo* nucleosome displacement, whereas at the latter it can further increase chromatin accessibility. Similar findings have been reported for SMAD1-containing complexes in the context of hair follicular differentiation (Adam et al., 2018), suggesting it is a common feature of the activated SMADs.

We have further taken advantage of the ATAC-seq datasets to infer changes in TF occupancy driven by NODAL/Activin signalling, both at a locus-specific and genome-wide level. By combining computational approaches with reporter assays, we have demonstrated the functional relevance of the DNA motifs adjacent to the SMAD2-binding sequences at the *Pmepa1* SBS (a closed, FOXH1-independent site) and at the *Wnt3* SBS (an open, FOXH1-dependent site). The technique of using ATAC-seq data for footprinting is still in development and two major challenges should be taken into consideration – the detection of the footprints themselves and the assignment of footprints to matching TFs. ATAC-seq footprint analysis, such as the Wellington algorithm is mostly based on adapting tools originally developed for DNase I data and a consensus ‘gold standard’ in the data processing workflow is yet to be achieved (Buenrostro et al., 2013; Li et al., 2019; Pranzatelli et al., 2018; Vierstra and Stamatoyannopoulos, 2016; Yan et al., 2020). Moreover, it is still not clear to what extent footprint detection is affected by Tn5 cleavage preferences (Tsompana and Buck, 2014). Consequently, coupling the bioinformatic analyses with an experimental validation is essential to be able to correctly interpret the results. Furthermore, it is becoming clear that many TFs do not leave significant footprints at their binding sites at all. Live-cell experiments have revealed that TFs have a highly dynamic binding behaviour, with residence times at target loci between 5 and 15 s (Sung et al., 2016). This behaviour has been described for the glucocorticoid receptor, OCT2 and SOX4 and the pioneer factor FOXA1 (Chen et al., 2014; Sung et al., 2014; Swinstead et al., 2016). Here, we have shown that FOXH1 itself does not leave an obvious footprint, although its binding site at the *Lefty1* SBS is part of a detectable footprint. Consequently, only those TFs with long residence times of binding to DNA, such as CTCF, generate detectable footprints in the ATAC-seq experiments using the Wellington algorithm. In contrast, the BaGFoot approach is more valuable, as it does not rely entirely on protection from DNA cleavage. Instead it detects changes in TF activity between conditions that include increased cleavage at nucleotides flanking the binding site (Baek et al., 2017).

The second challenge – identifying the TFs that match the footprints – is complicated by the fact that many TFs recognize highly similar motifs and a single TF can have multiple sequence preferences (Vierstra and Stamatoyannopoulos, 2016). The FOX motif footprinted at the *Pmepa1* SBS is a good example of such redundancy. Several members of the FOX TF family that recognize this particular FOX motif share extensive overlap in chromatin binding genome-wide (Chen et al., 2016). Consistent with this, our focused screen revealed that individual knockout of many of the FOX TFs expressed in P19 cells had no effect on *Pmepa1* NODAL/Activin-induced transcription, with the important exception of FOXO3 or FOXI3.

From the ATAC-seq data, we find that the footprints at the *Pmepa1* SBS are inducible, suggesting that these FOX TFs are not bound to the closed chromatin prior to signalling, but instead bind with the activated SMAD complexes, as shown for FOXH1 at the *Lefty1* and *Lefty2* enhancers (Coda et al., 2017). Members of the FOXO subfamily were previously shown to cooperate with SMAD2/3–SMAD4 to induce *CDKN1A* (also called *p21Cip1*) in epithelial cells in response to TGF-β, and FOXO3 has previously been shown to cooperate with the SMADs to regulate cyclin induction in ovarian cancer (Fu and Peng, 2011; Seoane et al., 2004). FOXI3, in contrast, represents a novel SMAD2/3 co-factor.

### Deciphering the mechanism responsible for delayed transcriptional activation downstream of NODAL/Activin signalling

One of our most striking findings from the BaGFoot analysis was that TCF/LEF family members show an increased occupancy at SBSs in response to prolonged NODAL/Activin signalling. This is consistent with previous studies, as during mesendoderm differentiation of mouse ESCs, NODAL and WNT signals cooperate to regulate the expression of developmental genes such as *T* and *Eomes* (Wang et al., 2017). In that case, however, the WNT ligand induction was shown to be downstream of p53 family members, and the possibility that *Wnt3* was a NODAL target gene, as we have shown here, was not addressed. Importantly, we also observed that the TCF/LEF1 motifs within the SBSs were occupied only after prolonged NODAL/Activin signalling. Coupling this observation with the fact that the expression of *Wnt3* is rapidly induced in response to NODAL/Activin signalling, we propose that the crosstalk between the SMADs and the TCFs is regulated via a so-called self-enabling mechanism. In this model, the SMAD-mediated expression of WNT3 triggers activation of the WNT pathway that signals to the LEF1 and/or TCF7L2 TFs that then serve as SMAD co-regulators of other genes (Fig. 7C). In a similar way, during mesendodermal differentiation of ESCs SMAD2, SMAD3 and SMAD4 cooperate with FOXH1 to induce the expression of the genes encoding LDTFs T and GSC, which then work in concert with activated SMADs to specify, respectively, mesoderm and endoderm fates at later stages of development (Faial et al., 2015; Teo et al., 2011). Our present findings now show that a self-enabling mechanism can coordinate SMAD interactions not only with some LDTFs, but also with the effectors of other signalling cascades.

When characterizing the cooperation between NODAL/Activin and WNT signals, our observation that the induction of *Wnt3* requires the simultaneous binding of activated SMAD complexes, FOXH1 and ZIC3 adds a further layer of complexity. ZIC3 is a member of the ZIC family of TFs and is involved in the regulation of differentiation processes under control of NODAL signalling (Herman and El-Hodiri, 2002; Houtmeyers et al., 2013; Purandare et al., 2002; Sutherland et al., 2013; Ware et al., 2006). More specifically, the ZIC3-binding site is also enriched in SMAD2 and FOXH1 ChIP-seq datasets obtained from human ESCs differentiated into endoderm (Yoon et al., 2011). Moreover, in a mouse model of X-linked heterotaxy, ZIC3 mutations cause severe cardiac defects only in conjunction with a NODAL haploinsufficiency (Ware et al., 2006) and, in humans, mutations in ZIC3 and the NODAL type II receptor ACVR2B are a common cause of heterotaxy and associated cardiovascular anomalies (Ma et al., 2012). In light of these studies, our results suggest that ZIC3, in concert with FOXH1, acts as the molecular switch that triggers the crosstalk between NODAL and WNT signals (Fig. 7C).

In conclusion, our work provides a framework to decipher the transcriptional network orchestrating long-term responses to NODAL/Activin signalling. We have demonstrated that activation of the NODAL/Activin–SMAD pathway induces dynamic changes in chromatin accessibility and in the binding activity of multiple TFs. We have shown that SMAD2 binding directly induces chromatin remodelling at closed chromatin sites, and we identified FOXI3 and ZIC3 as novel SMAD cofactors required for SMAD-dependent transcriptional regulation. Finally, we uncovered a self-enabling transcriptional cascade whereby NODAL/Activin signalling induces the expression of WNT3, which then activates the WNT-β-catenin-TCF signalling cascade, which cooperates with the activated SMADs to regulate the expression of NODAL/Activin target genes after prolonged signalling. Future work will aim to characterise the molecular mechanisms underlying the interactions of SMAD complexes with the cofactors identified in this study and investigate the crosstalk of SMAD complexes with the effectors of other signalling pathways whose binding activity changes in response to NODAL/Activin signalling.

## MATERIALS AND METHODS

### Cell line origin and authentication

P19 cells (Rudenko et al., 2013) were obtained from Grace Gill (Harvard Medical School). The cell line was banked by the Francis Crick Institute Cell Services, was certified negative for mycoplasma and validated as of mouse origin. Their identity was authenticated by confirming that the responses to ligands and the phenotype was consistent with published history. P19 cells and their derivatives were cultured in Dulbecco's modified Eagle's medium (DMEM; Thermo Fisher Scientific) containing 10% fetal calf serum (FCS; Sigma-Aldrich) (full medium).

### Generation of FOXH1 and ZIC3 knockout cell lines

To avoid problems of heterogeneity in the P19 population, we first isolated a clone of P19 cells that had the same characteristics as the P19 pool with respect to gene expression profiles and pSMAD2 induction patterns in response to Activin treatment. DNA oligonucleotides corresponding to the sgRNA sequences (see Table S2) targeting *Foxh1* or *Zic3* were cloned into pSpCas9(BB)-2A-GFP (PX458) (Ran et al., 2013), and the plasmids were transfected into P19 cells using Lipofectamine 2000 (Invitrogen) according to the manufacturer's instructions. At 48 h after transfection, the GFP-positive cells were FACS-sorted into 96-well plates. Resulting single-cell clones were screened by PCR using primers flanking the guide sites (see Table S2). The PCR fragments of positive clones were sequenced to identify the CRISPR/Cas9-mediated deletions/insertions. For FOXH1, we targeted the C-terminal SMAD-binding motif (Germain et al., 2000), and for ZIC3, we targeted the DNA-binding domain.

### Cell treatments and siRNA transfections

NODAL/Activin signalling was inhibited by overnight incubation with 10 μM SB-431542 (Tocris Bioscience), which was washed out three times with PBS prior to stimulation with 20 ng/ml Activin A (Peprotech) in full medium for different times. For the SB-431542 condition, after washout, cells were incubated for 1 h in 10 µM SB-431542 in full medium, providing a control for a possible transient effect of serum stimulation in the 1 h Activin samples. The untreated condition represents a chronic signalling state as a result of autocrine production of NODAL and GDF3 (Coda et al., 2017). CHIRON (Axon Medchem) and LGK974 (Cayman Chemical Company) were used at 5 µM, IWR (Sigma) was used at 20 µM. siRNA transfections were carried out using Lipofectamine RNAiMAX (Thermo Fisher Scientific) for 72 h at a final concentration of 20 nM. siRNAs were from Dharmacon and are listed in Table S2.

### Plasmid transfections, generation of stable cell lines, luciferase reporter assays and focused CRISPR screen

The pGL4.27-Pmepa1 SBS WT-luciferase reporter, the pGL4.27-Wnt3 SBS WT-luciferase reporter and their mutated versions were generated using the synthesised sequences listed in Table S2 cloned into the pGL4.27[luc2P/minP/Hygro] Vector (Promega). Note that in the pGL4.27-Pmepa1 SBS luciferase reporters there are three copies of the WT or mutant SBS sequences and in the pGL4.27-Wnt3 SBS luciferase reporters there are two copies of the WT or mutant Wnt3 SBS sequences. The plasmid encoding TK-Renilla was also from Promega.

Cells were transfected with the appropriate plasmids using Lipofectamine 2000 (Invitrogen) according to the manufacturer's instructions. The P19 line stably expressing the pGL4.27-Pmepa1 SBS WT-luciferase reporter together with TK-Renilla was generated by transfecting the cells with the appropriate plasmids together with a plasmid carrying the puromycin resistance gene (pSUPER-retro-puro; OligoEngine). Cells were then selected with 2 μg/ml puromycin (Sigma).

For the luciferase-based CRISPR-Cas9 screen, crRNA-tracrRNA duplexes targeting the different FOX transcription factors were mixed with 1.25 µg of recombinant Cas9 protein (TrueCut™ Cas9 Protein v2, Invitrogen) and electroporated using the Neon system (Invitrogen) with 1275 V, 30 ms pulse width and 1 pulse time. The Edit-R crRNA (Dharmacon) and Edit-R tracrRNA (U-002005-50, Dharmacon) used are listed in Table S2. At 48 h after plasmid transfection or gRNA-Cas9 complex electroporation, cells were treated with drugs or growth factors for the appropriate times and luciferase reporter assays were performed as previously described, using the Dual-Glo assay system (Promega) following the manufacturer's instructions (Levy et al., 2007).

### RNA extraction and qRT-PCR

Total RNA was extracted using Trizol (Thermo Fisher Scientific) according to manufacturer's instructions. cDNA synthesis and quantitative (q)PCRs were performed as described previously (Gronroos et al., 2012). Primer sequences are listed in Table S2. All qPCRs were performed with the PowerUp SYBR Green Master Mix (Thermo Fisher Scientific) with 300 nM of each primer and 2 µl of diluted cDNA. Fluorescence acquisition was performed on either a 7500 FAST machine or QuantStudio 12 Flex (Thermo Fisher Scientific). Calculations were performed using the ΔCt or ΔΔCt method, and levels of mRNA are expressed, respectively, as fold change relative to *Gapdh* mRNA or 8-h Activin-treated control cells. Unless otherwise stated in the figure legends, means±s.e.m. from at least three independent experiments are shown.

### DNA pulldown assays

DNA pulldown (DNAP) assays were performed as previously described with some modifications (Levy et al., 2007). Nuclear lysates were generated using extraction buffer containing 360 mM NaCl, and the DNAPs were performed in the presence of a 40 μg of non-biotinylated competitor mutant oligonucleotide to reduce non-specific binding. The oligonucleotides corresponding to WT and mutated *Pmepa1* and *Wnt3* SBSs are shown in the relevant figures and all oligonucleotide sequences are given in Table S2. Western blotting was carried out using standard methods. The list of the antibodies used is shown in Table S2.

### ATAC-seq sample preparation

Samples for ATAC-seq were obtained as described previously, with minor adaptations (Buenrostro et al., 2015). P19 cells were stimulated as appropriate and 100,000 cells were pelleted at 180 ***g*** for 5 min at 4°C. Pellets were washed once with 50 μl of cold PBS, and the cell suspensions were centrifuged at 180 ***g*** for 5 min at 4°C. To lyse the cells, samples were resuspended in 50 μl of cold lysis buffer (10 mM Tris-HCl pH 7.4, 10 mM NaCl, 3 mM MgCl_2_ and 0.1% IGEPAL CA-630) and immediately centrifuged at 500 ***g*** for 10 min at 4°C. Supernatants were then discarded and the nuclei gently mixed with 50 μl of transposition reaction mix. The transposition reaction was incubated at 37°C for 30 min, directly followed by a column purification step carried out using the PCR purification MinElute Kit (Qiagen). Here, 10 μl of Elution Buffer (Qiagen) were used for eluting the transposed DNA from the columns. To generate the libraries for next-generation sequencing, the transposed DNA fragments were amplified by PCR using the Veriti 96-well Thermal cycler (Applied Biosystems). Samples were then cleaned up with the PCR purification MinElute Kit (Qiagen) and eluted in 20 μl of Elution Buffer (Qiagen). Finally, an additional clean up step was performed using AMPure beads (Beckman Coulter, Inc.) to remove excess primers. Since the size of the library fragments provides a good indication of the success of the transposition reaction, the High Sensitivity DNA assay was performed on all samples using the 2100 Bioanalyzer (Agilent). For each biological replicate, two libraries were generated for each treatment condition to function as technical replicates, for a total of eight samples for each individual experiment. Upon Bioanalyzer analysis, for each biological replicate one sample per condition was picked amongst the technical replicates based on the fragment size distribution. A total of eight samples (four from each biological replicate) was then submitted for next generation sequencing.

### ATAC-seq – reads processing, filtering and alignments

The ATAC-seq experiment was performed in biological duplicate. Samples were prepared as described, and 51-bp pair end reads were generated on an Illumina HiSeq 2500. Raw reads from each sample were adapter-trimmed using cutadapt 1.9.1 with parameters ‘-a CTGTCTCTTATA -A CTGTCTCTTATA, minimum-length=25 – quality-cutoff=20’. BWA 0.6.2 (Li and Durbin, 2009) with default parameters was then used to perform genome-wide mapping of the adapter-trimmed reads to the mouse mm10 genome downloaded from the UCSC (Karolchik, 2004). Read group addition, duplicate marking, and insert size assessment was performed using, respectively, the picard tools AddOrReplaceReadGroups, MarkDuplicates and CollectMultipleMetrics (version 2.1.1.). Finally, reads mapped to mitochondrial DNA were removed using the pairToBed command from BEDTools 2.26.0-foss-2016b (Quinlan and Hall, 2010), and additional filtering was performed to remove read pairs that were discordant, mapped to different chromosomes, ambiguously mapped, had an insert size >2 kb, or mismatch >2 in any reads. All bam file sorting and indexing was performed with samtools 1.3.1 (Li et al., 2009). Replicate-level reproducibility across samples was assessed by counting read pairs that overlapped the union set of ATAC-seq peaks using the Subread featureCounts tool version 1.5.0 (Liao et al., 2014) with the parameters ‘- O, minOverlap 1, primary, ignoreDup -p -B -C – donotsort’. Read counts between replicates were then plotted on a log_10_ scale after quantile normalization using the ‘normalize.quantiles’ function in R version 3.3.1. When individual samples were combined to increase the read depth, the filtered alignments from each library were merged using the picard MergeSamFiles command. Duplicate marking was re-performed on the merged alignments, and they were subsequently filtered for duplicate reads, leading to 500–600 million reads for merged sample.

### ATAC-seq peak calling, generation of coverage tracks and IGV browser displays

Genome-wide ATAC-seq peaks and normalized BedGraph coverage tracks for the merged samples were obtained using MACS2 callpeak 2.1.1.20160309 (Zhang et al., 2008) with the parameters ‘gsize=mm, keep-dup all, nomodel, shift – 100, extsize 200 - B, SPMR, cutoff-analysis – broad’. The annotate Peaks.pl program from HOMER 4.8 (Heinz et al., 2010) was used to annotate ATAC-seq peaks relative to mm10 RefSeq features downloaded from UCSC. BedGraph coverage tracks generated by MACS2 were converted to BigWig using the bdg2bw utility available in the kent tools package from UCSC 20161115 (Kent et al., 2010), and visualized using the IGV genome browser. Finally, in order to obtain a union set of intervals, the ATAC-seq peaks from all samples were merged. For visualization of ChIP-seq data, SMAD2 tracks have been extended and smoothened as described previously (Coda et al., 2017).

### Overlapping of BED files and comparison of lists of elements

To intersect or subtract different BED files, BEDTools or the Table browser found within the UCSC Genome browser were generally used (Karolchik, 2004). To identify common elements within two, three or four different lists, the Venny tool was used (http://bioinfogp.cnb.csic.es/tools/venny/).

### DiffReps analysis and hierarchical clustering

The individual replicate samples were employed to call differential ATAC-seq sites between conditions. Intervals of differential ATAC signal were obtained using Diffreps 1.55.4 (Shen et al., 2013) with the parameters ‘window 200, step 20, nsd broad, frag 0, noanno, nohs’. To identify changes in chromatin accessibility at SBSs, the resulting files were intersected with the consensus ATAC-seq and SMAD2 peaks using the BEDTools. To increase the stringency of the data, we considered only the diffReps interval which had a padj lower then 0.1 and a log_2_FC greater than 0.5 as an absolute value. Hierarchical clustering using Euclidean distance was employed to group the different SBSs on the basis of the diffReps interval log_2_FC values.

### Footprint and genome-wide motif analyses

Footprint analysis was performed across the union set of ATAC-seq peaks on the unshifted, merged alignments using the ‘wellington_footprints.py’ command in the pyDNAse package 0.2.5 (Piper et al., 2013) with parameters ‘-fp 6,30,2 -fdr 0.05 -A’. Strand-specific coverage tracks representing Tn5 transposase cut sites were then generated using the pyDNAse command ‘dnase_wig_tracks.py’ with the ‘-A’ parameter, and visualised using the IGV genome browser. Finally, the individual footprints from all samples were merged to obtain a union set of footprint intervals.

To retrieve the genome-wide locations of all known motifs in the HOMER database, the ‘scanMotifGenomeWide.pl’ command was used with parameters ‘-bed -int -keepAll’. The resulting files were then intersected with the merged footprint intervals present in both ATAC-seq and SMAD2 peaks, using the BEDTools command intersectBed and setting the percentage of overlap to 10^−9^ (i.e. 1 bp). This analysis allowed calculation of motif footprint frequency, and the generation of different kinds of footprint heatmaps and plots for each of the known motifs in the HOMER database. First, motif centric heatmaps were obtained using the pyDNAse ‘dnase_to_javatreeview.py’ command with parameters ‘--window_size 100 -A -n’. Then, to identify changes in motif accessibility over time, differential heatmaps were generated by calculating the absolute value of the difference between each signalling condition compared to the SB-431542 sample. Additional differential heatmaps were also produced to conserve the strand information. This was achieved by extracting the absolute difference between each signalling condition and the SB-431542 sample, and then weighting it by ±1 depending on whether a change in sign was observed. Finally, the average profile plots around motif sites were generated using the pyDNAse ‘dnase_average_profile.py’ command with parameters ‘--window_size 100 -A’.

### FIMO analysis

The find individual motif occurrences (FIMO) analysis in Figs 3A and 6D was performed using as input the DNA sequence of the consensus SMAD2 peaks, which overlap an ATAC consensus peak (456), and the following motif matrices: GTAAACA for FOX and GGCCYCCTGCTGDGH for ZIC3. FIMO was run using default parameters. The FIMO analysis in Fig. 3B was carried out using as inputs the DNA sequence corresponding to the footprint F.3 within the *Pmepa1* SBS and the motif matrices generated by the BaGFoot package described above.

### BaGFoot analysis

Changes in TF activity between biological conditions in the experiment were assessed using the BaGFoot software package (version 0.9.6; Baek et al., 2017). Alignments were merged across replicates from the same condition to increase the coverage depth, and the analysis was performed relative to the MACS2 narrowPeak files generated for each of these conditions. BaGFoot was also run separately on the ATAC-seq peaks that were common to SMAD2 ChIP-seq peaks. The motif matrices required to run BaGFoot were generated by the ‘FimoAndMakeMotifList.R’ script provided by the authors of the package. Mappability files based on 51-mers of the mouse mm10 reference genome were created and provided to BaGFoot via the Mappability_Map (v1.0.0) tool available from the PeakSeq software (Rozowsky et al., 2009).

### Public availability of data

The ATAC-seq data have been submitted to the NCBI Gene Expression Omnibus (GEO) under the accession number GSE178315. The SMAD2 ChIP-seq dataset is the one generated and analysed in Coda et al. (2017) and is publicly available under the accession number GSE77488. The SMAD2 target gene categories are those previously identified and described (Coda et al., 2017).

### Statistical analysis

Statistical analysis was performed in Prism 8 (GraphPad). At least three independent experiments were performed for statistical analysis unless otherwise specified in the Figure legends. For qPCRs, statistics were performed using an unpaired two-tailed *t*-test.

## Supplementary Material

Supplementary information
