# Supplementary material for: A network of transcription factors governs the dynamics of NODAL/Activin transcriptional responses
Source: J Cell Sci. 2022 Apr 26;135(8):jcs259972. doi: 10.1242/jcs.259972 (PMC9080556; doi:10.1242/jcs.259972)
Supplement: Supplementary information [file joces-135-259972-s1.pdf]

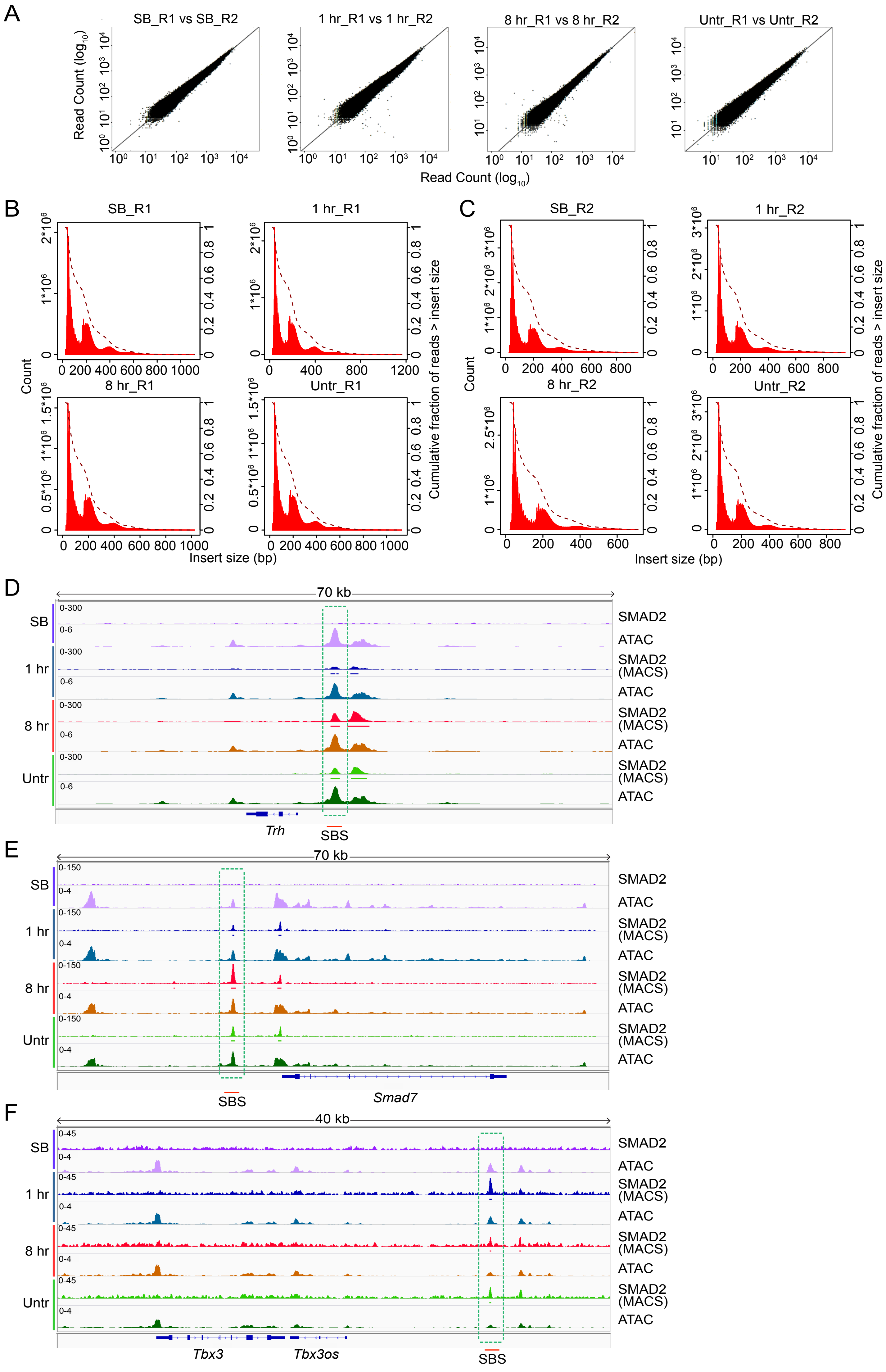
A
SB_R1 vs SB_R2
1 hr_R1 vs 1 hr_R2
8 hr_R1 vs 8 hr_R2
Untr_R1 vs Untr_R2
Read Count (log10)
Read Count (log10)
B
SB_R1
1 hr_R1
8 hr_R1
Untr_R1
Count
Cumulative fraction of reads > insert size
Insert size (bp)
C
SB_R2
1 hr_R2
8 hr_R2
Untr_R2
Count
Cumulative fraction of reads > insert size
Insert size (bp)
D
70 kb
SB
1 hr
8 hr
Untr
SMAD2
ATAC
SMAD2 (MACS)
ATAC
SMAD2 (MACS)
ATAC
SMAD2 (MACS)
ATAC
Trh
SBS
E
70 kb
SB
1 hr
8 hr
Untr
SMAD2
ATAC
SMAD2 (MACS)
ATAC
SMAD2 (MACS)
ATAC
SMAD2 (MACS)
ATAC
SBS
Smad7
F
40 kb
SB
1 hr
8 hr
Untr
SMAD2
ATAC
SMAD2 (MACS)
ATAC
SMAD2 (MACS)
ATAC
SMAD2 (MACS)
ATAC
Tbx3
Tbx3os
SBS

Fig. S1. Validation of the ATAC-seq data.

(A) Scatter plots showing the correlation between replicates for the four time points. Displayed are the normalized read counts for the ATAC-seq peaks identified by MACS.2 in each sample. (B and C) The insert size distribution of the ATAC-seq fragments is displayed as a continuous red line for each sample of either the first (B) or the second (C) biological replicate. The frequency of insert size is also reported on all plots as a dotted red line. 1 hr, 1 hr Activin; 8 hr, 8 hr Activin; SB, SB-431542; Untr, Untreated; R1, Replicate 1; R2, Replicate 2.

(D–F) IGV browser visualization of SMAD2 ChIP-seq and ATAC-seq experiments performed in P19 cells treated as indicated. For the SMAD2 ChIP-seq the MACS-called peaks are also shown. The genomic loci displayed refer to the delayed gene *Trh* (D), the ‘transiently induced’ gene *Smad7* (E) and to the ‘repressed’ gene *Tbx3* (F). The green dotted box indicating the SBSs denotes a site at which SMAD2 binds to open chromatin.

Journal of Cell Science • Supplementary information

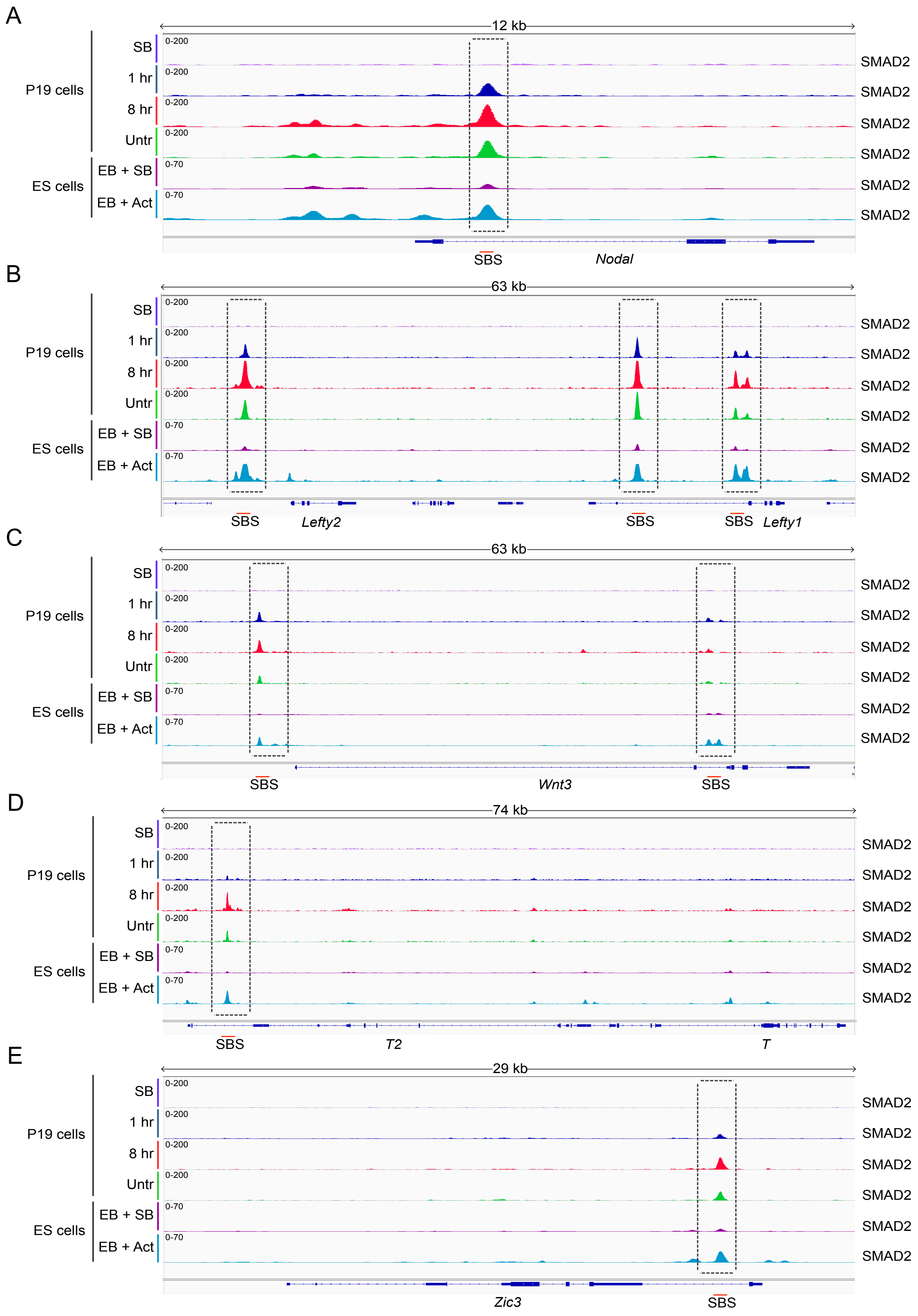

Journal of Cell Science • Supplementary information

Fig. S2. SMAD2 ChIP-seq peaks are conserved in mouse embryoid bodies.
(A–E) IGV browser visualization of SMAD2 ChIP-seq from P19 cells treated as indicated compared to the SMAD2 ChIP-seq tracks obtained from Aragón et al 2019 (*32*). Note that the SMAD2 ChIP-seq peaks in response to Activin are completely conserved between P19 cells and mouse embryoid bodies. The genomic loci displayed refer to the representative genes *Nodal* (A), *Lefty1* and *Lefty2* (B), *Wnt3* (C), *T* (D), *Zic3* (E). SB, SB-431542; Act, Activin; 1 hr, 1hr Activin; 8 hr, 8 hr Activin; Untr, untreated; EB, embryoid body

Journal of Cell Science • Supplementary information

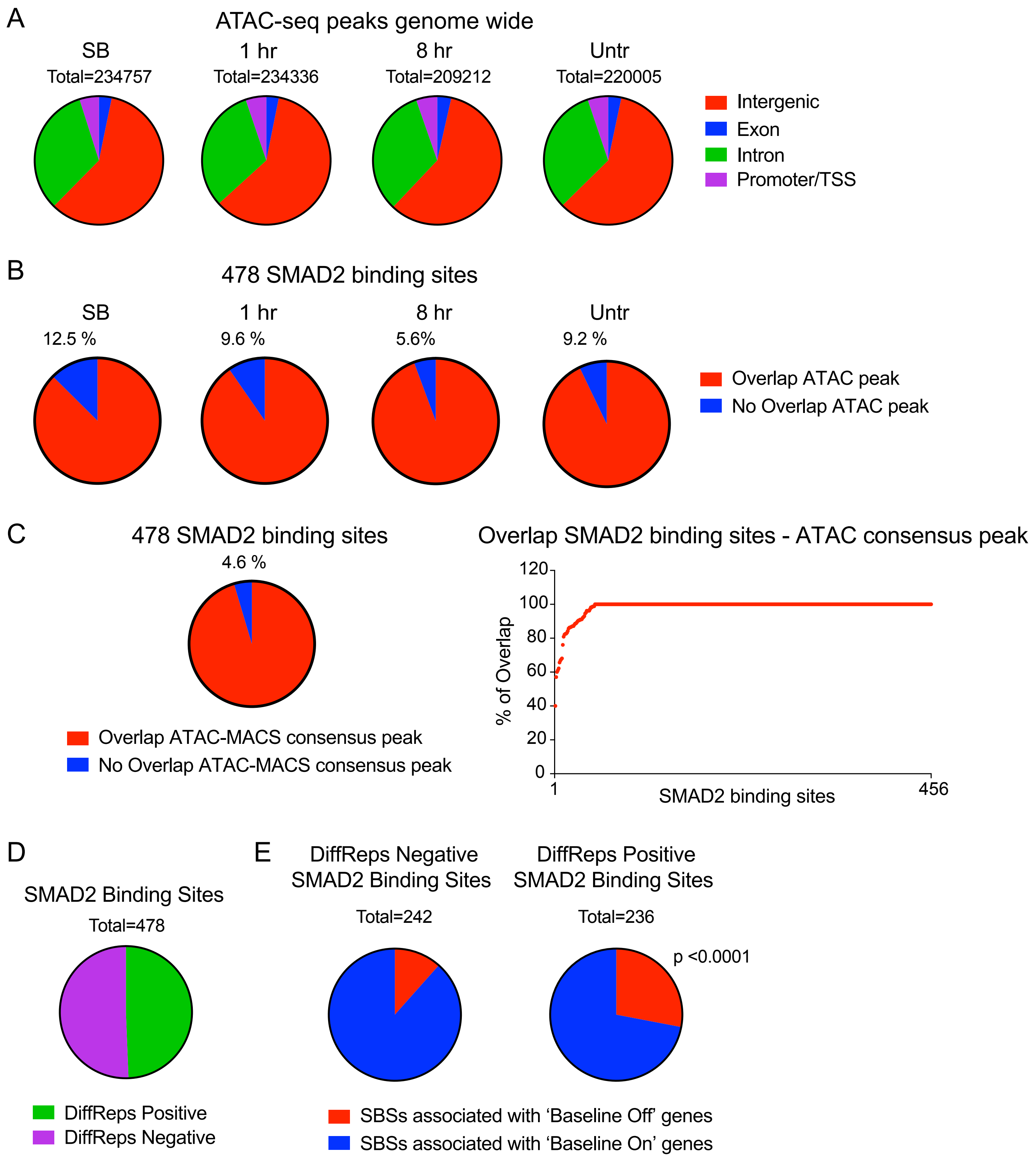

Journal of Cell Science • Supplementary information

Fig. S3. The SMAD2 binding peaks overlap with ATAC-seq peaks

(A) MACS.2 software was used to call ATAC peaks and assign them to either intergenic, exon, intron or promoter/TSS DNA regions. For each sample the total number of peaks and their distribution according to the four genomic features used for the annotation is reported.

(B) For each of the 478 SMAD2 binding consensus intervals, the distance to the closest ATAC-seq peak was computed for all signaling conditions. For each sample, the fraction of SMAD2 binding sites overlapping or not with an ATAC-seq interval for at least 1 bp is displayed in red or blue respectively.

(C) Left panel, the portion of SBSs which overlapped with a DiffReps interval in at least one signaling condition is displayed in red, whilst the fraction of DiffReps negative SBSs is shown in blue. Right panel, for each of the 456 SBSs positive for intersection, the portion of the SMAD2 interval overlapping the corresponding ATAC peak is plotted. On the graph, the SBSs on the x axis are displayed in ascending order according to their percentage of overlap.

(D) The 478 consensus SMAD2 peaks were divided into two groups based on a change in chromatin accessibility in at least one signaling condition.

(E) For each group, the percentage of SBSs associated with 'baseline off' (red) or 'baseline on' (blue) genes is indicated. Un-paired Chi-square test was performed on the data using a 95% confidence interval and the resulting p-value is reported on the graph.

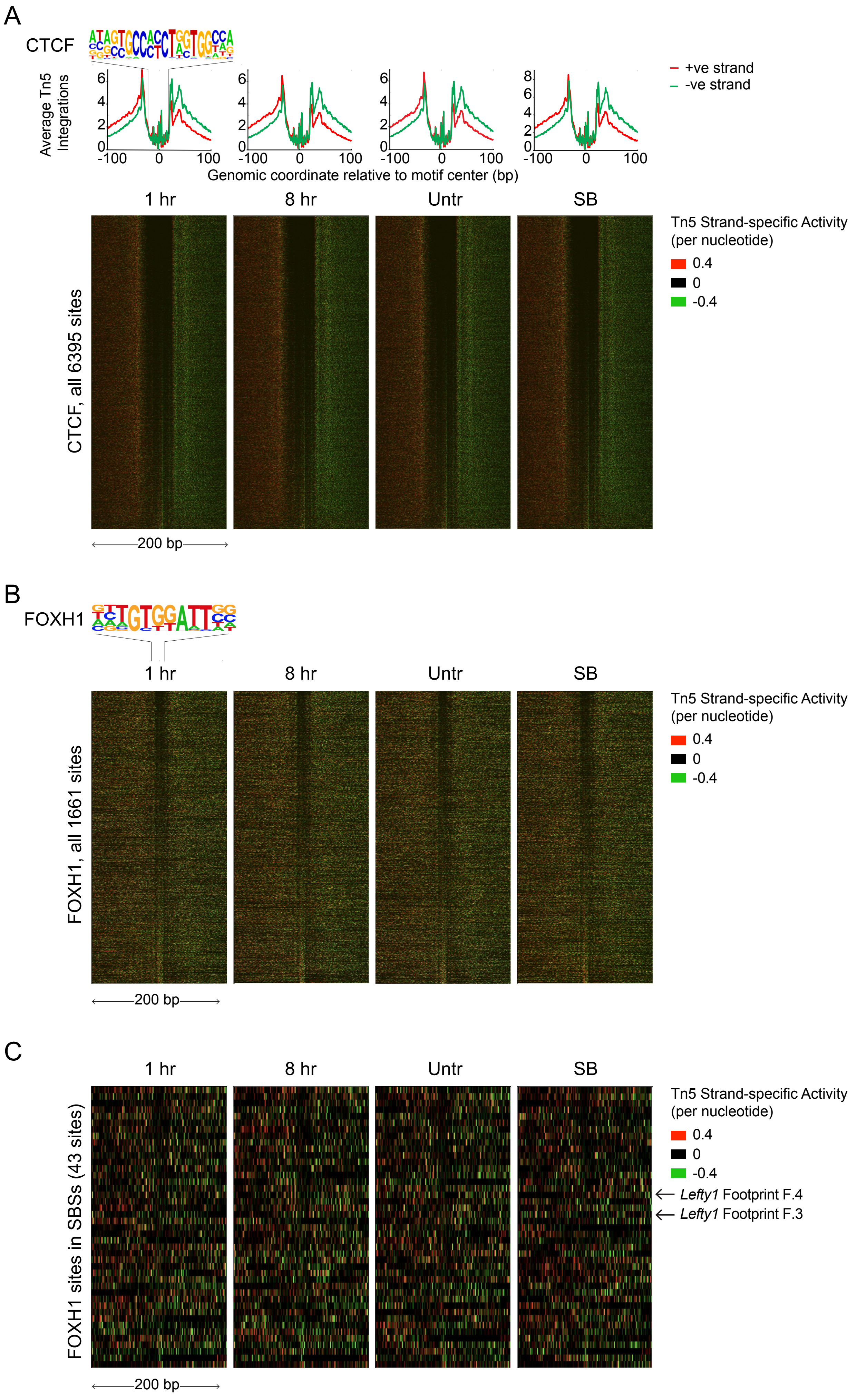

Journal of Cell Science • Supplementary information

Fig. S4. In contrast to CTCF, FOXH1 binding does not result in a detectable footprint. (A) Heatmaps showing the per nucleotide Tn5 cutting activity for each site with a CTCF binding motif intersecting a consensus footprint interval genome wide for the four timepoints/ conditions. The data refer to a 200 bp window centered on the CTCF motif. An excess of positive strand Tn5 cuts over the negative strand is shown in grades of red, the opposite is indicated in grades of green. In the 1 hr Activin heatmap, the order of the sites from top to bottom reflects their decreasing Footprint Occupancy Score as calculated by the Wellington algorithm. For all the other samples, sites are instead ordered accordingly to the 1 hr Activin plot, thus each row refers to the same locus moving across the signaling conditions. On top of each heatmap are also displayed the aggregate Tn5 cleavage profiles as obtained from averaging Tn5 integrations in the corresponding samples, with the CTCF motif logo from the Homer database shown on top of the first plot.

(B) As for (A), but for the FOXH1 binding motif genome wide. The FOXH1 motif logo from the Homer database displayed on top.

(C) As in (B), but for FOXH1 binding motifs intersecting a consensus footprint interval under a SMAD2 binding site. The arrows indicate the FOXH1 sites associated with the *Lefty1* SBS footprints F.3 and F.4 (Figure 2A) detected in the presence of NODAL/Activin.

1 hr, 1 hr Activin; 8 hr, 8 hr Activin; Untr, untreated; SB, SB-431542.

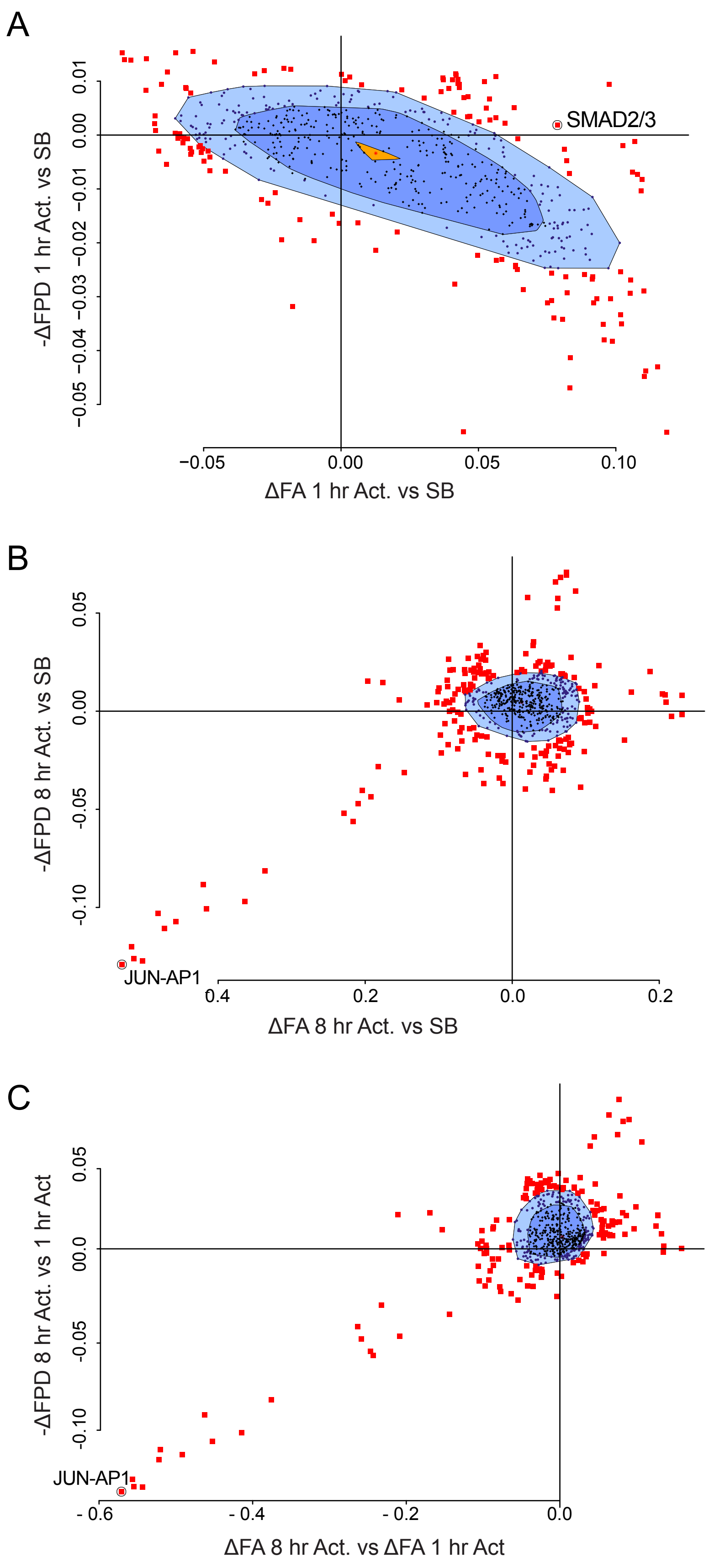

Fig. S5. BaGFoot plots showing ΔFA and ΔFPD for all the ATAC-seq peaks in P19 cells after different treatments.

(A) 1 hr Activin compared to SB-431542 (SB).

(B) 8 hr Activin compared to SB-431542.

(C) 8 hr Activin compared to 1 hr Activin.

Journal of Cell Science • Supplementary information

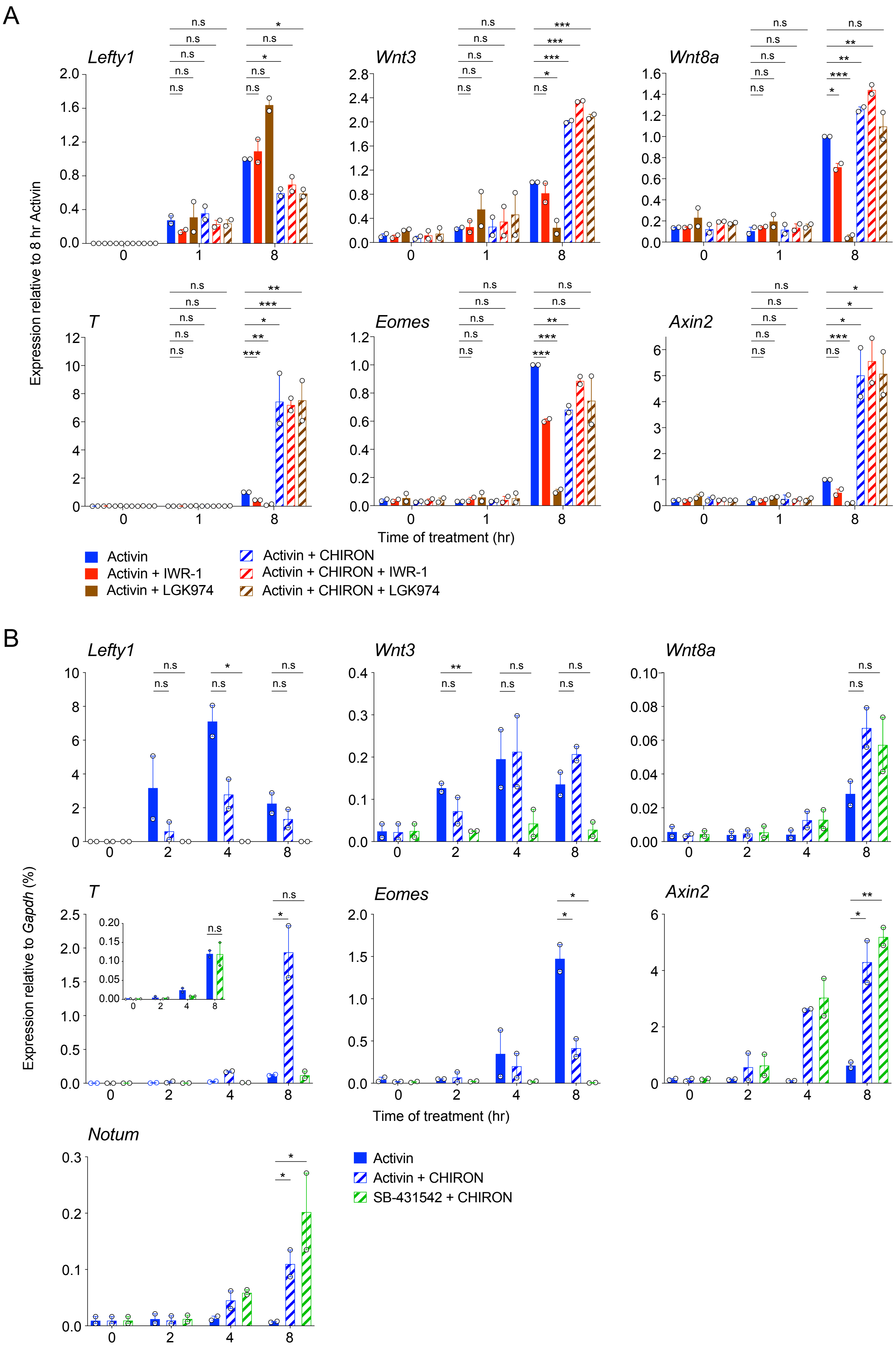

Journal of Cell Science • Supplementary information

Fig. S6. *T* and *Eomes,* but not *Lefty1,* require WNT signaling for their induction as well as NODAL/Activin.

(A) Cells were treated overnight with SB-431542, washed out, then either treated with SB-431542 for 1 hr or with Activin for the indicated times. The WNT/β-Catenin pathway inhibitors IWR or LGK974 were added as indicated (solid bars). Alternatively, the WNT/β-Catenin pathway activator CHIRON (5 μM) was added alone or in combination with IWR or LGK974 (dashed bars). qPCR was performed for the genes shown.

(B) Cells were treated overnight with SB-431542, washed out, then stimulated for the indicated times with Activin alone (solid blue), or in combination with CHIRON (blue dashes). Alternatively, cells were treated with SB-431542 in combination with CHIRON (green dashes). qPCR was performed for the genes shown. For *T,* a blow up is shown to better compare the Activin and SB-431542 + CHIRON conditions.

In A and B means and SEM are plotted for two independent experiments performed in triplicate of gene expression values normalized to *Gapdh* values. n.s, not significant; *, p value of <0.05; **, p value of <0.01; ***, p value of <0.001; ****, p value of <0.0001.

Journal of Cell Science • Supplementary information

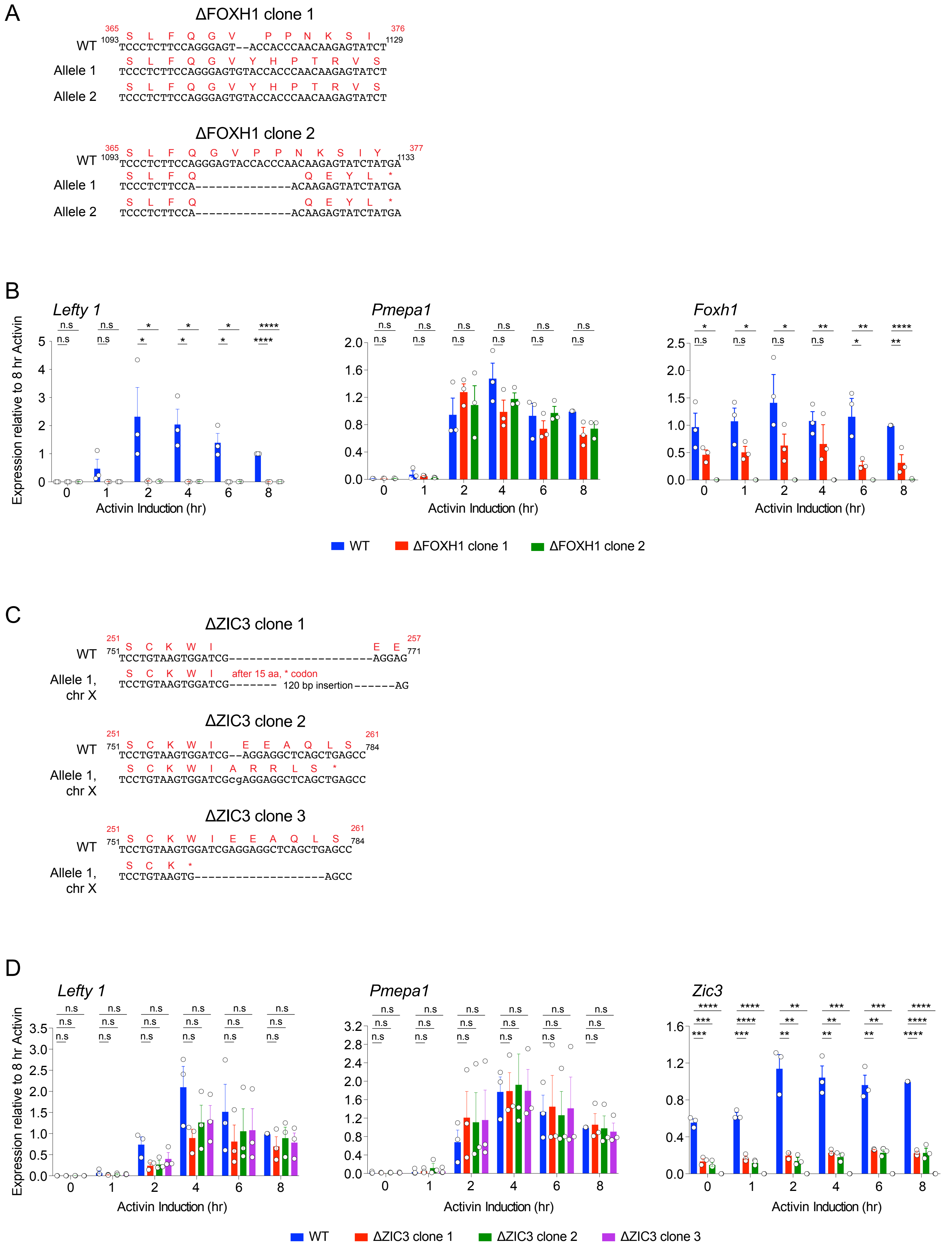

Journal of Cell Science • Supplementary information

Fig. S7. Characterization of the P19 ΔFOXH1 and P19 ΔZIC3 clones.

(A) Sequences of FOXH1 in the regions around the guides in P19 ΔFOXH1 clone 1 and clone 2. From the sequencing we conclude that there are two alleles of FOXH1. The protein sequence for the wild type is shown in red above the DNA sequence. A 2 bp insertion is evident in clone 1, whilst clone 2 is characterized by a 14 bp deletion. Both these clones are homozygous.

(B) A time course of Activin induction was performed for the indicated times on two independent clones with deletions for FOXH1, and on WT P19 cells as a control. Plotted are the means and SEM of three independent experiments performed in duplicate.

(C) Sequences of ZIC3 in the regions around the guides in P19 ΔZIC3 clone 1, clone 2 and clone 3. *Zic3* is on the X chromosome and as P19 cells are male, there is only one allele . The protein sequence for the wild-type (WT) is shown in red above the DNA sequence. Clone 1 has a 120-bp insertion, clone 2, a 2-bp insertion and clone 3 a 19-bp deletion.

(D) A time course of Activin induction was performed for the indicated times on three independent clones with deletions for ZIC3, and on WT P19 cells as a control. Plotted are the means and SEM of three independent experiments performed in duplicate. n.s, not significant; *, p value of <0.05; **, p value of <0.01; ***, p value of <0.001; ****, p value of <0.0001.

Table S1. A list of the SBSs that showed temporal changes in ATAC-seq. The ATAC-seq values (log2FC relative to the SB condition) at different times after Activin stimulation are displayed along side the SMAD2 ChIP-seq peaks normalised reads (log2FC relative to the SB condition).

Click here to download Table S1

Table S2. Key Resources Table

Click here to download Table S2

Journal of Cell Science • Supplementary information
